# SecAntiGami: A Secondary-Antibody-Inspired RNA Origami for Qualitative Aptamer Validation

**DOI:** 10.1021/acs.nanolett.6c03022

**Published:** 2026-10-30

**Authors:** Linus S. Frey, William Verstraeten, Mai P. Tran, Dana Venkert, Luca Monari, Cody Geary, Kerstin Göpfrich

**Affiliations:** † Center for Molecular Biology of Heidelberg University (ZMBH), 9144 Heidelberg University, Berliner Str. 45, D-69120 Heidelberg, Germany; ‡ Biophysical Engineering Group, Max Planck Institute for Medical Research, Jahnstraße 29, D-69120 Heidelberg, Germany; § Cluster of Excellence SynthImmune, Heidelberg University, Im Neuenheimer Feld 344, D-69120 Heidelberg, Germany

**Keywords:** RNA origami, RNA aptamers, kissing loops, dynamic light scattering, aptamer validation, giant unilamellar lipid vesicles (GUVs)

## Abstract

RNA aptamers are short, single-stranded RNA molecules that fold into specific three-dimensional structures and selectively bind molecular targets. Despite applications in therapeutics, diagnostics, and biosensing, tools for aptamer validation remain scarce compared to well-established pipelines for other biomolecular binders such as antibodies. Here, we introduce SecAntiGami—a secondary-antibody-inspired RNA origami—as a modular tool for signal enhancement and aptamer characterization. SecAntiGami binds aptamers of interest through programmable kissing-loop interactions and carries a fluorescent aptamer for visualization. It amplifies aptamer-dependent size increases in dynamic light scattering assays and enables fluorescence-based aptamer localization. We rationally design three different variants of a biotin-binding aptamer and use SecAntiGami to demonstrate that all of them retain their functionality, enabling efficient integration of aptamers into RNA origami structures. SecAntiGami serves as a versatile tool for qualitative aptamer validation for synthetic biology, structural RNA nanotechnology, RNA therapeutics, and diagnostics.

## Graphical abstract

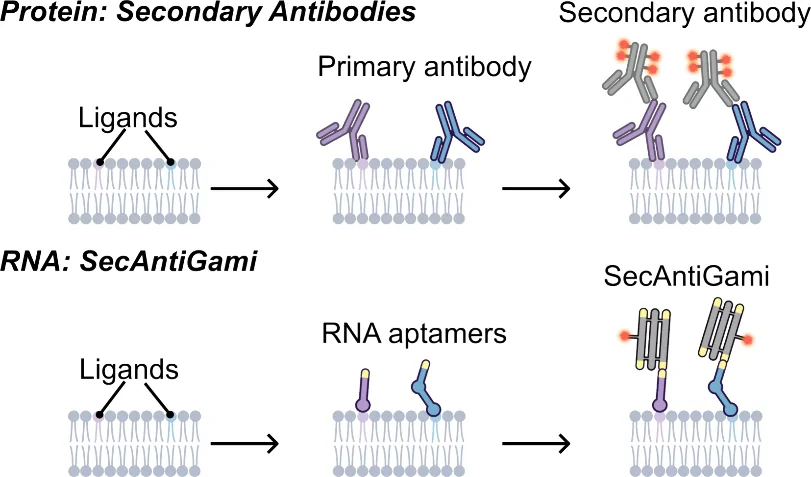

RNA exhibits a diverse range of functions from catalysis mediated by ribozymes^1,2^ to molecular recognition through RNA-based binding motifs called aptamers,^3–9^ extending far beyond its canonical function in the central dogma of molecular biology. RNA aptamers in particular offer exciting therapeutic potential, with the US aptamer market projected to grow at a compound annual growth rate of over 24% through 2030 and two aptamers having received regulatory approval.^10,11^ Nature provides many highly specific aptamer sequences for binding metabolites,^12,13^ or other targets.^3,9^ In addition, *in vitro* selection of aptamers by Systematic Evolution of Ligands by EXponential enrichment (SELEX)^14–16^ and similar techniques^4,6,17,18^ enable aptamer discovery through selection and enrichment from large pools of randomized sequences.

*In vitro* selection is a complex and labor-intensive process^14,15^ that requires screening of 10^12^ to 10^15^ random sequences and testing successive truncations of candidates, often followed by other significant postselection optimizations to yield tight binders.^19^ The limited tools available for the identification, characterization, and screening of aptamer variants is a roadblock to the rapid development of new RNA functions. Throughout this work, we use *validation* to denote the qualitative confirmation that a candidate aptamer binds its target, *characterization* to denote the description of this binding behavior and the effect of the aptamer’s structural context, and *screening* to denote the comparison of multiple candidates or variants in order to distinguish binders from nonbinders. AI tools are beginning to accelerate aptamer discovery,^20–24^ further increasing the demand for experimental testing of their binding properties.

Currently, surface plasmon resonance (SPR) is the gold standard in the field for aptamer characterization. It detects small changes in refractive index upon binding of the aptamer to a ligand, and allows for quantification of the binding affinity and kinetics.^25,26^ However, SPR is highly time-consuming, material-intensive and expensive. Dynamic light scattering (DLS), in turn, measures the increase in particle size by detecting changes in the diffusion constant. While DLS has commonly been used to verify nanoparticle functionalization,^27^ to our knowledge, it has not been used to characterize binding of free aptamers to ligand-presenting nanoparticle surfaces, likely because DLS is not sensitive enough to detect a size increase caused by the binding of small and flexible aptamers. Other methods, such as zeta-potential^28,29^ and gel electrophoresis^30^ measurements provide a complementary readout for nucleic acid adsorption on nanoparticles, however, ion concentrations must remain within a well-defined window,^31,32^ not necessarily compatible with aptamer screening under native conditions.

Although observation of aptamer-ligand binding via fluorescence microscopy would be possible by addition of a fluorophore or a fluorescent aptamer to the RNA aptamer of interest, both of these strategies could interfere with the folding of the unknown secondary structure and thereby its function.^33,34^ This is because fluorescent molecules are often hydrophobic, causing covalently linked nucleic acids to interact with them in certain configurations.^35^ Likewise, fluorescent light-up aptamer motifs typically fold into complex quadruplex structures that can easily interfere with the folding of a linked aptamer. Thus, a simple, inexpensive, and programmable alternative method for assessing and designing aptamers for membrane and particle surface interactions is needed.

Unlike RNA aptamers, antibodies are frequently characterized in conjunction with secondary antibodies.^36–38^ Secondary antibodies are typically labeled with enzymes, radioactive markers, or fluorophores to generate or amplify signals through binding to primary antibodies that are specific to the target molecule (Figure 1A). This two-step detection system allows the use of a single labeled secondary antibody with a wide range of primary antibodies.^38^ In contrast, RNA does not possess a naturally conserved domain, analogous to the Fc region in primary antibodies, that can be recognized by a common secondary antibody. Instead, modular RNA detection has generally relied on engineered scaffolded recognition elements that require adding a custom RNA handle, rather than using a universal intrinsic motif.^39,40^ Our approach follows this route: each target aptamer is equipped with a short, complementary kissing-loop (KL) handle that the SecAntiGami recognizes. The analogy to secondary antibodies is therefore one of function and workflow—a single, reusable, labeled reagent that reports on many different primary binders—rather than one of label-free universality, since some aptamer-specific design remains necessary (as discussed below). Taking inspiration from secondary antibodies, we use RNA origami to amplify the binding signal upon aptamer-target recognition. RNA origami^41^ is a versatile method in RNA nanotechnology that can be used to design specific structures^42,43^ that can imitate or manipulate cell functions.^44–47^ RNA origami is produced by cotranscriptional folding from a DNA template, making it scalable and well-suited for biotechnological applications.^41^ We deliberately choose RNA origami rather than a simpler DNA nanostructure, such as a DNA tile or a multivalent DNA probe, for three reasons. First, the scaffold and its fluorescent reporter are produced in a single step by cotranscriptional folding from one DNA template, so that no covalent dye conjugation or postsynthesis purification is required. Second, visualization relies on a genetically encoded light-up aptamer, providing label-free fluorescence, whereas a DNA-based probe would still require a covalently attached fluorophore. Third, RNA–RNA kissing-loop interactions provide a programmable and orthogonal handle for recognizing RNA aptamers that is designed automatically with ROAD.^42^ We note that the size-amplification function alone could also be achieved with a multivalent DNA probe; the advantage of RNA origami lies in this integrated, label-free, and scalable design, which is furthermore compatible with *in situ* transcription in the presence of vesicles or cells.

**Figure 1:**
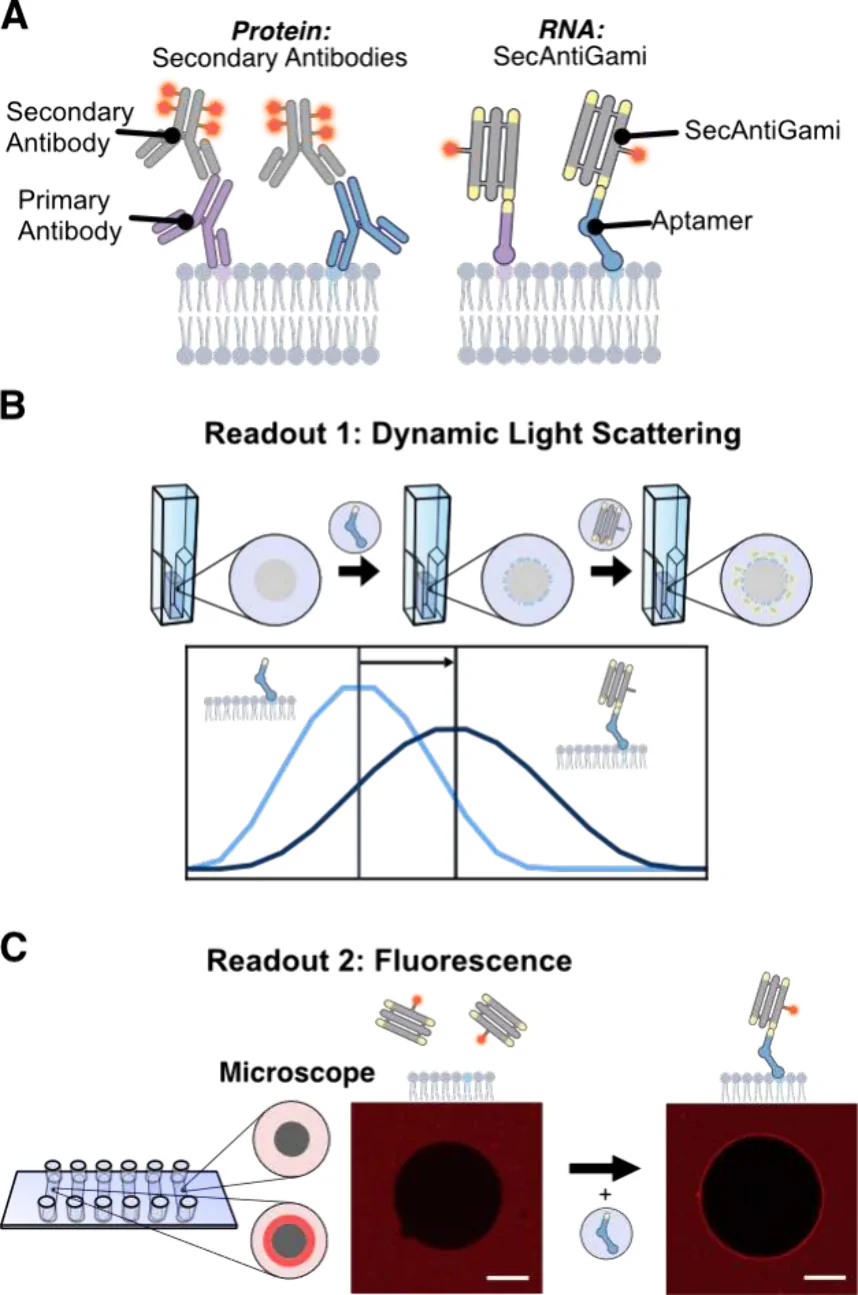
SecAntiGami as a tool for aptamer validation. (A) Secondary antibodies can be used to create or amplify the binding signal of primary antibodies (left). The RNA-based (Sec)­ondary (Anti)­body-inspired RNA Ori­(Gami) (SecAntiGami) creates and amplifies the binding signal of RNA-based binding motifs, known as aptamers (right). (B) Readout 1: A small dynamic light scattering (DLS) signal arising from direct aptamer–vesicle binding is enhanced by the SecAntiGami. (C) Readout 2: Binding of an aptamer to a vesicle is visualized with confocal fluorescence microscopy. The signal is generated by dye binding to the light-up aptamer sequence in the SecAntiGami, eliminating the need for expensive chemical modification. Scale bars: 50 μm.

We generate a specific RNA origami, namely the (Sec)­ondary (Anti)­body-inspired RNA Ori­(Gami) construct, termed “SecAntiGami”, which contains multiple RNA–RNA binding sites for binding to target aptamers. SecAntiGami is designed as a secondary-antibody-inspired RNA origami scaffold for modular aptamer labeling (Figure 1A). It acts as a signal amplifier that enhances detection of aptamer binding by DLS and enables signal localization by fluorescence microscopy (Figure 1B, C, see Video S1 for a broadly accessible video animation of the SecAntiGami concept). Because the fluorescence originates from a light-up aptamer that binds a dye, it eliminates the need for expensive covalent chemical modifications that are typically required for antibody labeling.^48^ SecAntiGami can be rapidly produced by *in vitro* transcription from a DNA template as required. Compared with existing tools, SecAntiGami thus provides a fast, low-cost, and label-free qualitative (binder/nonbinder) readout of aptamer–target recognition through two independent and accessible modalities: a size increase measured by DLS, and fluorescence accumulation measured by microscopy, the latter also applicable in and on cells. This complements, rather than replaces, quantitative methods such as SPR, which yield binding affinity and kinetics but are slower and more resource-intensive. We envision that the use of SecAntiGami will facilitate the functional validation of RNA aptamers, offering a versatile and straightforward method for assessing and designing aptamers for targeted molecular interactions.

We design the SecAntiGami (Figure 2A, B) using the RNA Origami Automated Design software (ROAD).^42^ The design consists of three parallel RNA double helices, interconnected by dovetails and internal kissing loop (KL) interactions.^49,50^ The SecAntiGami is produced by *in vitro* transcription with a T7 polymerase from a 355 nucleotide long double-stranded DNA template and folds during transcription at 37 °C (all sequences and corresponding gel analyses are provided in Tables S1–S2 and Figures S1–S2). A malachite green aptamer, i.e. a specific RNA sequence with a submicromolar affinity to the triphenylmethane dye malachite green (MG),^7,51^ is positioned at the 3′ end of the SecAntiGami. Upon binding, the malachite green aptamer (MGA) increases the fluorescence of the dye > 2000-fold^7,51^ and thus allows for fluorescence visualization of the RNA origami without covalent modification with a fluorophore. Since the 3′ end of the RNA is released last during transcription, fluorescence is indicative of complete transcription. Indeed, we can track the SecAntiGami production as an increase in fluorescence in plate reader experiments, where we observe saturation after 8 h of transcription (Figure S3). With atomic force microscopy, we verify the correct assembly of the SecAntiGami (Figure S4). The resulting images show particles that match the expected dimensions of the RNA origami, with a width of approximately 6 nm and a length of 15 nm.

**Figure 2:**
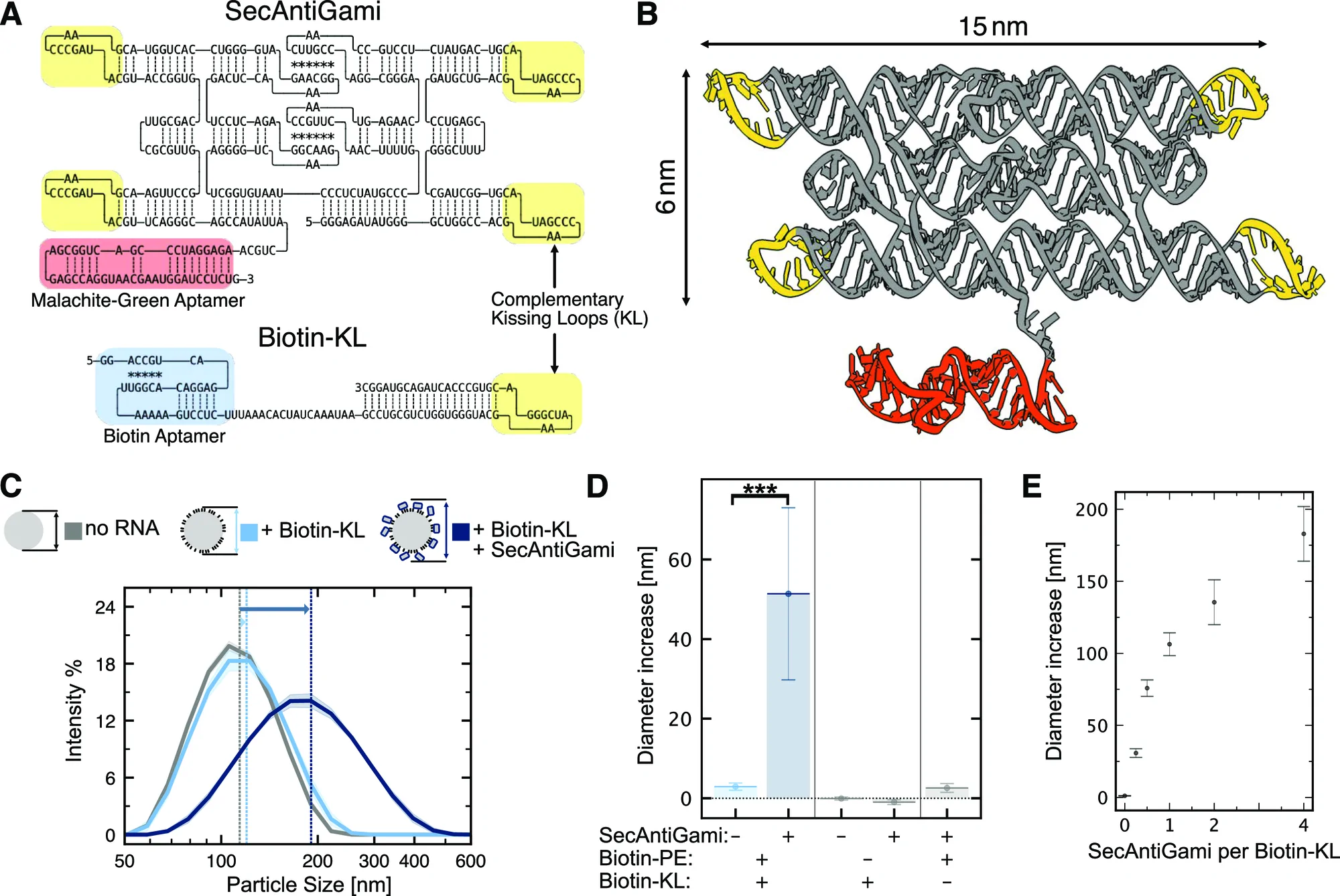
SecAntiGami design and validation. (A) Top: Secondary structure blueprint of the SecAntiGami design with its four kissing loops (KLs) for aptamer binding (highlighted in yellow) and the malachite green aptamer (red). Bottom: Secondary structure blueprint of the target aptamer with the KL (yellow) complementary to the SecAntiGami. Here, we use a biotin aptamer (highlighted in blue). (B) Three-dimensional structure representation of the SecAntiGami (side view). (C) Particle size distributions obtained from DLS experiments with biotinylated small unilamellar vesicles (SUVs, gray), biotinylated SUVs incubated with the Biotin-KL aptamer (light blue), and biotinylated SUVs incubated with the Biotin-KL aptamer in the presence of the SecAntiGami (dark blue). The vertical dashed lines show the mean size of the respective distributions; the shaded area corresponds to the standard error of the mean (*n* = 3). The arrows indicate the diameter increase plotted in (D). (D) Diameter increase in the presence (+) or absence (−) of the SecAntiGami, biotin-PEG-functionalized lipids (biotin-PE), and Biotin-KL aptamers. Three independent experiments are shown (mean ± SEM); the horizontal lines indicate the means of all independent repeats. Statistics: Welch’s *t* test (*n* = 3), significance with p-values < 0.001 are marked with ***. (E) Diameter increase as a function of the SecAntiGami per Biotin-KL aptamer. Two independent experiments, with three technical repeats each, are shown (*n* = 2, mean ± SEM).

The crucial design feature of the SecAntiGami is its capacity to bind to the aptamer of interest, analogous to the binding to a primary antibody. This is achieved by four terminal 180° KLs at the edges of the SecAntiGami design (Figure 2A, highlighted in yellow). In parallel, the target aptamer is equipped with a stem loop that is complementary to these KLs. These KLs form a sequence-specific six base pair loop-to-loop contact between the SecAntiGami and the target aptamer. We validate this binding using agarose gel electrophoresis (Figure S5), which shows that the addition of aptamer to the SecAntiGami results in a mass and charge increase of the RNA complex.

Note that the secondary structures of aptamers are often unknown and frequently sensitive to the neighboring sequences (Figure S6). Since the sequence of the KL-containing stem comprises only a single duplex, its sequence can be designed to be orthogonal to the aptamer sequence. We note that such sequence orthogonality reduces, but does not by itself guarantee, the absence of any effect on aptamer folding; we therefore verify *in silico* that the appended KL leaves the predicted secondary structure of each construct unchanged (Figure S7). This strategy separates the folding of the SecAntiGami scaffold and the target aptamer. Additionally, the SecAntiGami and the aptamer of interest can be folded independently. This allows the aptamers to be folded into their native secondary structure more easily. Embedding the aptamer within a large RNA origami or inserting a light-up aptamer or fluorescent label to the aptamer can greatly impact its folding (Figure S6).^35^ Furthermore, embedding multiple copies of a large aptamer into a single RNA construct creates a bottleneck for DNA template synthesis,^52^ often resulting in failed synthesis of the gene, whereby SecAntiGami circumvents this common limitation as well as reducing synthesis costs.

In order to establish a proof of concept and to showcase that the SecAntiGami can be used for signal enhancement, we equip a known biotin-binding aptamer^8^ (Figure 2A, bottom) with a KL that binds to the SecAntiGami (referred to as Biotin-KL). This aptamer was previously shown to effectively bind to biotinylated membranes.^44,47,53^ We prepare small unilamellar lipid vesicles (SUVs) from a lipid mix containing DOPC and 5% biotin-PE. In DLS experiments, we observe that incubation of SUVs with the Biotin-KL aptamer results in a very small increase in diameter (Figure 2C), highlighting that faithful recognition of aptamer-target binding by DLS requires signal amplification. When the SecAntiGami is added, we indeed observe a significant (*p* < 0.001) diameter increase (Figure 2D). Moreover, this increase is specific to the presence of a biotin-binding aptamer. In the absence of the biotin-PE ligand or in the absence of the aptamer, the addition of the SecAntiGami has a negligible effect on the measured SUV size (Figure 2D). The fact that binding of the aptamer alone is nearly undetectable with DLS likely originates from the small size of the aptamer consisting of only 100 nucleotides in combination with the flexible PEG chain on the biotinylated lipid.

To gain further insight into the signal amplification characteristics of the SecAntiGami, we conduct a titration experiment, in which different SecAntiGami concentrations are added to a sample of SUVs with 5% biotin-PE lipids incubated with Biotin-KL RNA (Figure 2E). The successive addition of SecAntiGami reveals a dependency of the size increase on the SecAntiGami-to-aptamer ratio. We estimate that the maximum expected diameter increase due to binding of SecAntiGami to SUVs is approximately 80 nm (calculations in Table S3). However, the calculated maximum diameter increase is already obtained when we add 0.5 to 1 mol of SecAntiGami per mol of Biotin-KL aptamer (Figure 2E). Further addition of the SecAntiGami results in an increase of the original diameter of approximately 200 nm compared to the SUVs without RNA, indicating a SecAntiGami-mediated clustering of SUVs as a cause for the efficient signal enhancement. This clustering can originate from the multivalency of the SecAntiGami with its 4 KLs. In line with this hypothesis, a variation of the SecAntiGami with only one KL (Figure S8) shows a size increase of only 20 nm with lower variations compared to the diameter increase caused by the SecAntiGami with 4 KLs (Figure S8). Nevertheless, we adhere to the design with 4 KLs for aptamer testing due to the beneficial larger signal amplification. As a consequence of this pronounced ratio-dependency and the clustering of SUVs, the DLS size increase serves as a qualitative binder/nonbinder readout and cannot be used for quantitative comparisons of binding affinity between aptamers; a qualitative assessment, however, remains fully possible. If SUV clustering is undesirable, the ratio of SecAntiGami to aptamer can be maintained at 1 or lower.

We further study the time dependence of the diameter increase (Figure S9). The data suggest that a plateau is reached approximately 50 to 60 min after addition of the SecAntiGami. Accordingly, to achieve robust DLS signal amplification, we use a 1:1 SecAntiGami:aptamer ratio and incubate samples for 50 to 60 min for further experiments.

The purpose of the SecAntiGami strategy is to rapidly screen multiple aptamer sequences or variants for their binding function. As a proof-of-principle, we design three new variants of the known biotin aptamer. We position the aptamer in different orientations by modifying the connections between the core pseudoknot and the remainder of the RNA construct as illustrated in Figure 3A–D. These structural variants of the biotin aptamer allow us to test if the aptamer can be fused to RNA origami in different positions, such as internally where a free 5′ or 3′ end is not available, and to determine which, if any of the new orientations are capable of binding to biotin. Such structural variants of aptamers are useful to provide versatility in RNA design, e.g. to avoid repeated sequences which are difficult to synthesize, to position multiple aptamers in one strand or when the 5′ and 3′ ends are occupied for another functionality. Multiple aptamers on an RNA origami scaffold are of interest for therapeutic applications to enable multivalency, multitarget and improved avidity.^54^

**Figure 3:**
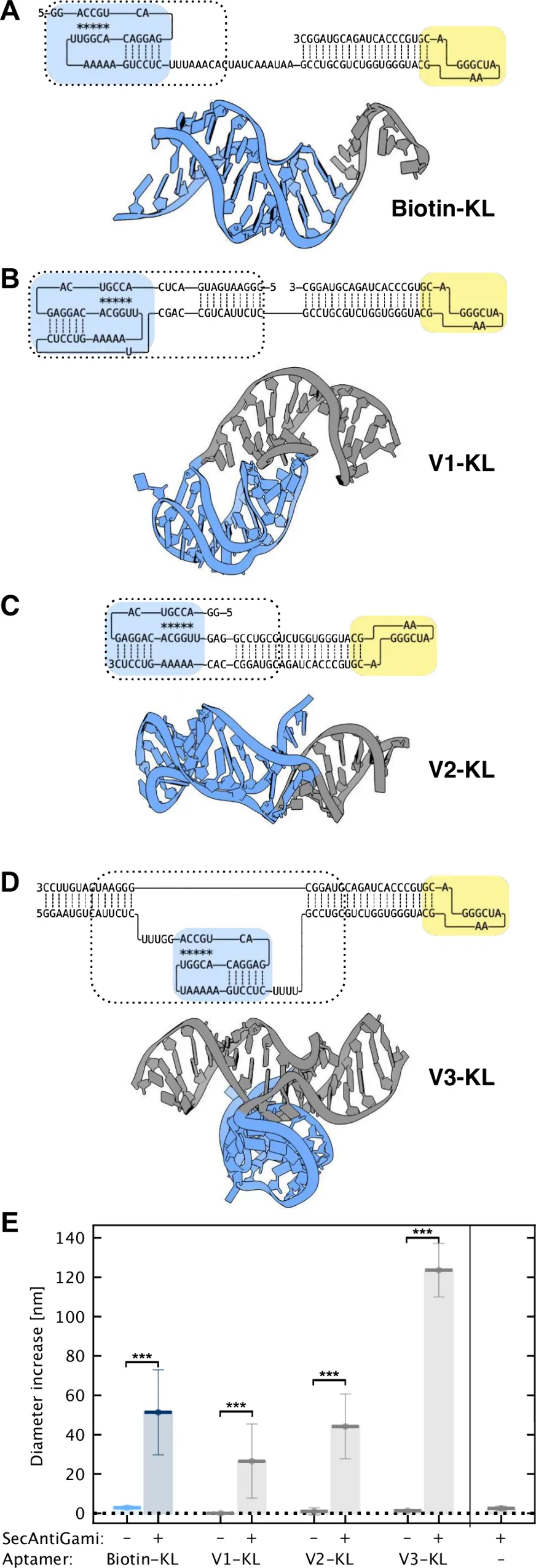
Design and validation of three new biotin aptamer variants. (A–D) Secondary structure blueprint of the Biotin-KL construct (A) and three new aptamer variants (B–D, biotin aptamer in blue). All are linked to the same KL sequence (yellow) and therefore compatible with the SecAntiGami shown in Figure 2A and B. For each model, the results of a coarse-grained folding simulation in SimRNA are shown for the dotted region of each blueprint, depicting how the aptamer variants are joined to the construct in different orientations. (E) Diameter increase in DLS experiments for the aptamer variants in the presence (+) or absence (−) of SecAntiGami. Three independent experiments are shown (mean ± SEM); horizontal lines indicate the means of all independent repeats. Statistics: Welch’s *t* test (*n* = 3), significance with p-values < 0.001 are marked with ***.

We again evaluate the binding of the aptamer variants to biotin-containing model membranes with SecAntiGami by DLS (Figure 3E). All three newly designed variants exhibit a pronounced ability to bind to SUVs containing biotinylated lipids. The attachment of the core aptamer pseudoknot at the end of a closed stem like in variant 1 (V1-KL) demonstrates variable binding efficiencies. Nevertheless, an attachment to a stem at the termini of the sequence like in variant 2 (V2-KL) and in the middle of a stem as in variant 3 (V3-KL) exhibit a robust binding signal, consistently detectable across all experiments. As an additional negative control, we create a biotin aptamer in the original orientation (like in Biotin-KL) but with a completely modified core binding sequence (Figure S8c). In accordance with the destruction of the binding motif, we observe no binding signal, showcasing the identification of a nonfunctional aptamer.

In addition to its applications in DLS experiments, the SecAntiGami can also be employed for fluorescence visualization of aptamer binding thanks to its integrated MGA, mimicking fluorescently labeled secondary antibodies. Fluorescence visualization can be particularly useful when testing aptamers in or on living cells. As shown in Figure 4A, the binding of aptamers to the membrane of giant unilamellar vesicles (GUVs) is directly seen on confocal images because the fluorescent SecAntiGami is concentrated at the surface of the vesicles. The fluorescence intensity on the GUV membrane is significantly increased in comparison to the control without Biotin-PE lipids (*p* < 0.001, Figure 4B). The negative control, in the absence of the aptamer, demonstrates that neither the fluorophore nor the SecAntiGami exhibits an inherent affinity for the membrane. Therefore, fluorescence accumulation on the membrane indicates successful binding of the tested aptamer in complex with the SecAntiGami.

**Figure 4:**
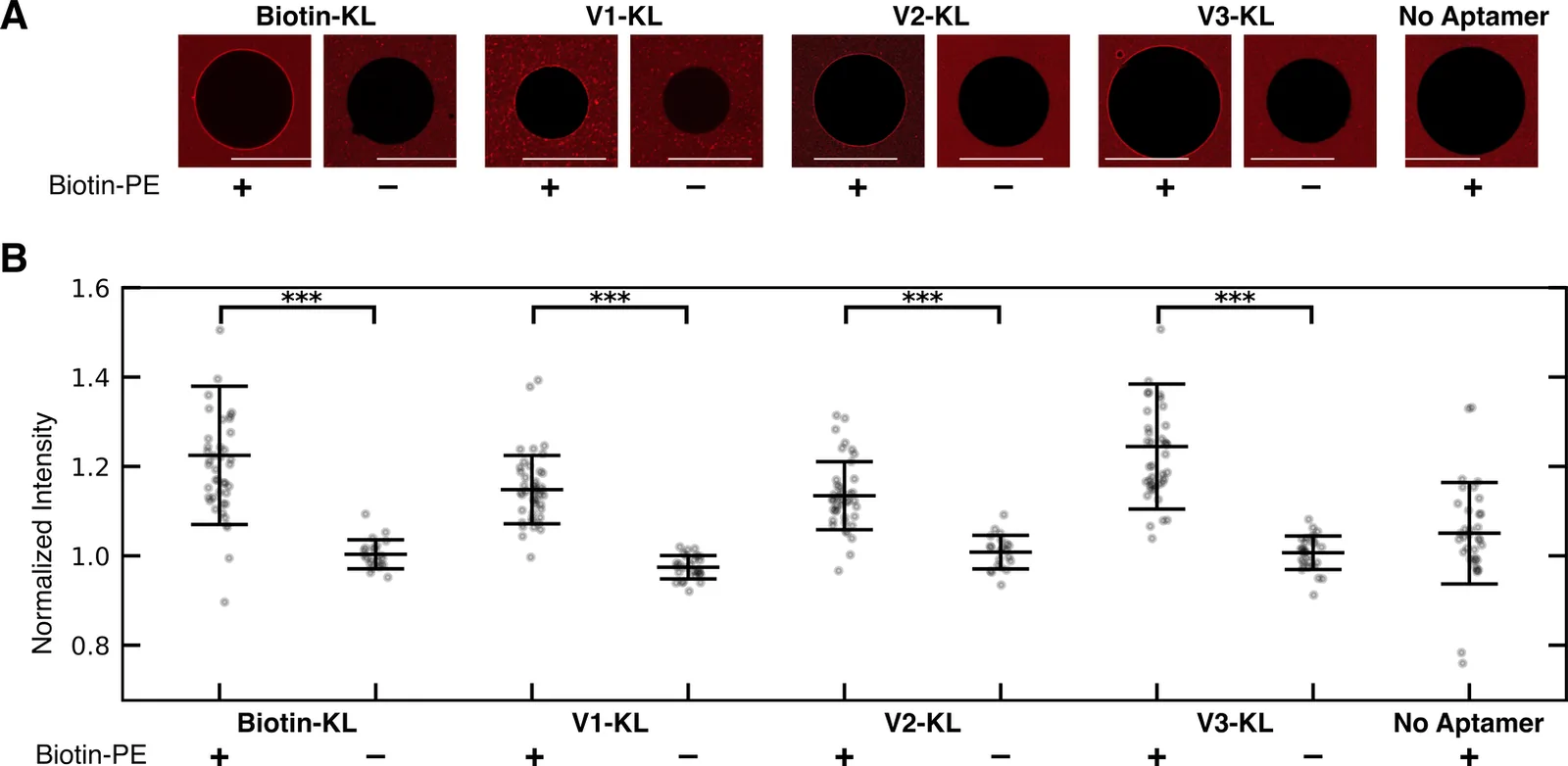
SecAntiGami as a fluorescent reporter for aptamer binding. (A) Confocal fluorescence images of representative biotinylated giant unilamellar vesicles (GUVs) with the Biotin-KL and three new aptamer variants in the presence of SecAntiGami for fluorescent reporting (red, SecAntiGami is labeled using malachite green aptamer and malachite green dye, λ_*ex*_ = 640 nm), scale bars: 50 μm. (B) Background-normalized fluorescence intensity on the GUV membrane for *n* = 22–44 GUVs in the presence (+) and absence (−) of biotin-PE lipids for the known biotin aptamer, its three new variants, and a “No Aptamer” control (mean ± SD). Statistics: unpaired two-sample Welch’s *t* test (*n* = 22–44), significance with p-values < 0.001 are marked with ***.

We next ask whether SecAntiGami can also be used with aptamers targeting larger proteins.^55^

To test this, we study the binding of a GFP aptamer to GUVs using confocal microscopy. GUVs are first functionalized with His-tagged GFP using Ni-NTA lipids (see Materials and Methods).^56^ We find that the fluorescence signal from SecAntiGami at the membrane is significantly increased relative to the negative control lacking the GFP-KL construct, confirming successful binding of the aptamer to the GUV reported by SecAntiGami (Figure S10). Together with the biotin system, this demonstrates that SecAntiGami reports binding for two structurally distinct classes of target—a small molecule and a protein—without any change to SecAntiGami itself, consistent with recognition occurring through the KL handle rather than through the aptamer’s target. A systematic evaluation across a graded series of binding affinities is an important next step; because the multivalent readout is qualitative (see above), such quantitative benchmarking would be best performed alongside an affinity method such as SPR.

To further highlight the modularity of SecAntiGami, we replace the MGA with the Broccoli aptamer (BrA) to yield a Broccoli SecAntiGami variant (Figure S11). As with the MGA/MG pair, the DFHBI-1T fluorescence increases significantly when bound by the BrA.^57^ When adding the Broccoli-SecAntiGami and Biotin-KL to GUVs, the fluorescence intensity on the GUV membrane is again significantly increased compared to GUVs without biotin (*p* < 0.001, Figure S11). Note that without SecAntiGami, there are limited options for fluorescence labeling of the biotin aptamer since the sequence of biotin aptamer is complementary to a variety of fluorescent aptamers (Figure S6). This also illustrates why we favor the modular SecAntiGami over the conceptually simpler alternative of directly fusing the aptamer of interest to a light-up aptamer such as the MGA. For a single, well-characterized aptamer, such a fusion is a reasonable and simpler route, and it would avoid vesicle clustering by reporting binding through fluorescence rather than size. However, light-up aptamers fold into structured, often quadruplex, motifs, and the biotin aptamer is partially complementary to the Broccoli, iSpinach, and Mango aptamers (Figure S6), so a direct fusion risks disrupting the fold of either module. The modular design circumvents this by folding the reporter and the aptamer independently and joining them noncovalently through a short KL. It also preserves the key secondary-antibody advantage of reusability—a single SecAntiGami reports on many different aptamers, each requiring only a short KL—and it avoids the DNA-synthesis bottleneck of long fused constructs,^52^ while additionally enabling the DLS readout.

We here introduce SecAntiGami, a secondary-antibody-inspired RNA origami that improves the qualitative evaluation of aptamer–target binding. The three-dimensional structures of aptamers are typically unknown and their folding can be sensitive to neighboring sequences. Therefore, the attachment of binding motifs and fluorophores for detection purposes can have unintended influence on the fold and therefore the target binding properties of the aptamer. We circumvent this problem with SecAntiGami: Its KL stem is completely paired and its sequence can be easily customized to not interfere with the aptamer sequence and its fold. SecAntiGami can be employed as a binding signal enhancer^58^ in DLS size measurements and as a fluorescent marker in microscopy. These properties render it an inexpensive and versatile qualitative tool for the development, identification, and testing of new RNA binding motifs in a scalable and time-efficient manner.

Like many assays for the characterization of primary antibodies via secondary antibody binding such as ELISA, we chose to immobilize the binding target of the aptamer of interest on a surface. In our study, we use lipid vesicle surfaces with potential applications in synthetic cell research and drug delivery. However, it is also conceivable to immobilize the target molecule on other surfaces such as beads or planar surfaces. Note that for planar surfaces, DLS cannot be used but fluorescence detection would still be possible.

Although SecAntiGami establishes a modular strategy for aptamer labeling, its current implementation is not fully universal in the manner of a conventional secondary antibody. Because binding relies on a compatible kissing-loop interface, some aptamer-specific design remains necessary. Moreover, the present readouts are primarily qualitative and do not yet provide a comprehensive quantitative assessment of performance. Careful quantification of the SecAntiGami’s analytical behavior will be essential for using it to identify the best-performing aptamers within a library.

In conclusion, we demonstrate that the SecAntiGami serves as a signal amplifier, facilitating detection of aptamer binding by DLS or fluorescence microscopy. Owing to its mechanism, the SecAntiGami is applicable to any aptamer-characterization assay that reports changes in fluorescence or size.

We envision that SecAntiGami could be used to enrich the pool of aptamers and their variants for biomedical uses or synthetic cell development. Future versions of SecAntiGami could be customized to incorporate additional or different fluorescent aptamers which allow for multiplexing SecAntiGami for different targets in parallelized assays. In addition to the potential for novel aptamer investigation, future development could transfer the use of SecAntiGami as an RNA-based reporter to other, conventionally antibody-based fields. Ultimately, SecAntiGami enables the rapid prototyping and validation of novel aptamer variants with diverse structural configurations, expanding the toolkit for RNA bioengineering and synthetic biology.

## Supplementary Material







## Data Availability

The data sets generated and analyzed during the current study are available from the corresponding author on reasonable request.

## Notes

Additional experimental details and calculations, materials and methods, and all DNA sequences (PDF)
DNA Sequences used in SecAntiGami: Secondary antibody-inspired RNA origami for aptamer validation (XLSX)
Video S1: SecAntiGami movie (MP4)
