# Supplementary material for: SecAntiGami: A Secondary-Antibody-Inspired RNA Origami for Qualitative Aptamer Validation
Source: Nano Lett. 2026 Oct 30;26(44):15250–8. doi: 10.1021/acs.nanolett.6c03022 (PMC13761691; doi:10.1021/acs.nanolett.6c03022)
Supplement: Supplementary file 1 [file nl6c03022_si_001.pdf]

Supplementary Information

SecAntiGami: A Secondary Antibody-Inspired RNA Origami for Qualitative Aptamer Validation

Linus S. Frey,[†,‡,§] William Verstraeten,[†,§] Mai P. Tran,[†] Dana Venkert,[†,¶] Luca Monari,[†] Cody Geary,[*,†,¶] and Kerstin Göpfrich[*,†,‡,¶]

†*Center for Molecular Biology of Heidelberg University (ZMBH), Heidelberg University, Berliner Str. 45, D-69120 Heidelberg, Germany*

‡*Biophysical Engineering Group, Max Planck Institute for Medical Research, Jahnstraße 29, D-69120 Heidelberg, Germany*

¶*Cluster of Excellence SynthImmune, Heidelberg University, Im Neuenheimer Feld 344, D-69120 Heidelberg, Germany*

§*These authors contributed equally.*

E-mail: c.geary@zmbh.uni-heidelberg.de; k.goepfrich@zmbh.uni-heidelberg.de

Materials & Methods

SecAntiGami design

All sequences used for the constructs presented in this work were designed using the RNA Origami Automated Design software (ROAD).[1]

In detail, a blueprint of the target structure was set up. The starting nucleotides and all other known sequences (aptamers and KL sequences) were fixed. The ROAD software was then used to generate 10 to 100 attempts for sequence design. The sequence with the lowest ensemble diversity was chosen and evaluated with RNAfold[2] and forna[3] as a part of the ViennaRNA web services.[4] Finally, a DNA template was designed with the T7 promoter sequence, such that the desired RNA sequence could be produced upon in vitro transcription. All DNA template and primer sequences can be found in Tables S1 and S2.

DNA amplification

The SecAntiGami DNA template was purchased from Twist Bioscience HQ. All other DNA templates and all primers were purchased from Integrated DNA Technologies. Prior to transcription, the DNA templates were amplified by PCR using Phusion High-Fidelity DNA Polymerase and HF-buffer (New England Biolabs, NEB) with an annealing temperature of 62 °C in 45 cycles. The resulting PCR products were analysed by agarose gel electrophoresis shown in Figure S1. For the GFP aptamer, two single stranded DNA strands were ordered and annealed (DNA dissolved in nuclease-free water to 10µM, then mixed and heated up to 85°C. Stepwise temperature reduction by -1°C per minute down to 20°C). No PCR was performed here. All DNA samples were stored at −20 °C until further use.

In vitro transcription of the SecAntiGami

The PCR-amplified DNA sequences were subjected to in vitro transcription (IVT) overnight at 37 °C using the HiScribe T7 High Yield RNA Synthesis Kit (NEB) and 5 ng µL^{-1} DNA

template. The transcribed RNA was stored at 4 °C for a maximum of 6 h. As a rough estimation of RNA concentration after IVT, part of the IVT was treated with $0.2\,\mathrm{U\,\mu L^{-1}}$ DNase I-XT (NEB) for 15 min at 37 °C and then purified using the Monarch Total RNA Miniprep Kit (NEB. Cat. No. T2010S). The RNA was eluted in nuclease-free water, and its concentration was quantified using an Implen NanoPhotometer NP80. It was assumed that the purified portion of the IVT reaction contained the same concentration of RNA as the unpurified part. The unpurified RNA from the IVT was used for DLS experiments.

Atomic force microscopy (AFM)

AFM imaging was performed using a NanoWizard 2 high-speed atomic force microscope (Bruker) in AC fast imaging mode, equipped with FASTSCAN-D cantilevers (Bruker Nano Inc., Camarillo, CA, USA; $f = 110$ kHz, $k = 0.25\ \mathrm{N\,m^{-1}}$). The imaging substrate was mica (diameter: 0.95 cm; Science Services GmbH, Munich, Germany; cat. no. E71856-01), glued into a small petri dish (Corning, diameter:35 mm, height: 10 mm). The IVT sample was imaged once (n=1), with two aliquots (0.5 μL each) of the IVT reaction deposited onto the mica substrate. No purification of the IVT samples was performed prior to imaging. To image the RNA origami, freshly cleaved mica was covered with imaging buffer (1× TAE, 20 mM $MgCl_2$). Subsequently, 1 μL of the in vitro transcription (IVT) sample was gently pipetted onto the mica surface. All AFM images were pre-processed, to remove imaging artefacts, with the open-access software Gwyddion[5] using standardized Align Rows and Levelling tools and then exported into SVG file format.

SUV preparation

All lipids were purchased from Avanti Polar Lipids, except 1,2-Dioleoyl-sn-glycero-3- phosphoethanolamine labeled with ATTO 488 (ATTO 488-PE) was purchased from ATTO-TEC GmbH. The following compounds were dissolved in chloroform: 1,2-dioleoyl-sn-glycero-3-phosphocholine (DOPC), 1,2-distearoyl-sn-glycero-3-phosphoethanolamine-N-[biotinyl(polyethylene glycol)-

2000] (Biotin-PE) and ATTO 488-PE. The dissolved and undissolved lipids were stored at −20 °C until further use.

For negative control SUVs, 100 mol% DOPC was used (DOPC-SUVs). Biotin-PE SUVs were prepared with 5 mol% of the biotinylated lipid and 95 mol% DOPC. The solvent was evaporated from the lipid mixtures while they were rotated under a gentle nitrogen flow in a glass vial. The lipid films were then dried under vacuum for 30 min and subsequently hydrated with 500 µL of biotin aptamer binding buffer (100 mM KCl, 10 mM HEPES, 5 mM $MgCl_2$). To facilitate the hydration process, the mixtures were incubated at 900 RPM for 10 min. Following hydration, the solution contains a lipid concentration of 2 mM. Subsequently, the mixtures were subjected to an extruding process utilizing a mini-extruder, with one filter support on each side (both supplied by Avanti Polar Lipids) and a Nuclepore Track-Etched membrane (Whatman) with a pore size of 100 nm. The SUVs were stored at 4 °C until use for up to 1 week.

Dynamic light scattering (DLS)

For DLS experiments, a Malvern Panalytical Zetasizer Nano ZS ZEN3600 DLS instrument was used in conjunction with polystyrene semi-micro cuvettes (Sarstedt, order number: 67.742). The instrument was configured to measure the particle size in a 173° backscatter mode with automatic measurement duration. The data was recorded at 25 °C with water as the dispersant (refractive index: 1.330) and LUV as the particle material (refractive index: 1.420; absorption: 0.000). Each single measurement of a sample was conducted in triplicates. Two independent experiments were conducted for the titration of SecAntiGami, three independent experiments for the majority of the other measurements, and three experiments for the Biotin-KL on Biotin-PE SUVs shown in Figures 2 and 3 in the main text. The size was measured in the same sample on consecutive occasions: initially, prior to the addition of RNA; subsequently, following the addition of aptamers; and finally, following every addition of SecAntiGami. In general, samples were incubated approx. 50 min to 60 min at room

temperature after addition of RNA. This time was decreased to 20 min for the additions of SecAntiGami during the titration.

The SUVs were diluted in the biotin aptamer binding buffer[6] (100 mM KCl, 10 mM HEPES, 5 mM $MgCl_2$) to a final lipid concentration of 100 µM and a volume of 300 µL. The addition of aptamer or SecAntiGami was equivalent to 600 pmol of RNA. This differed only in the titration experiments, where 600 pmol of aptamer was used, and the amount of SecAntiGami was increased from 150 pmol to 2.4 nmol in the sample during the measurement. The intensity-weighted primary output was employed for all analytical purposes.

Statistical analysis of DLS and microscope experiments

For the data analysis, a Python code was set up with the aid of ChatGPT, the code is available on the group's GitHub page `https://github.com/Biophysical-Engineering-Group`. In detail, a mean value μ of each measurement and the corresponding standard deviation σ (Eq. 1) were calculated from the technical replicates.

$$\sigma = \sqrt{\frac{1}{N-1}\sum_{i=1}^{N}(x_i-\mu)^2} \tag{1}$$

In this context, x_i represents the value of the respective replicate, while N denotes the number of replicates. The standard deviation σ is calculated by dividing by $N-1$, given that only a limited sample size is employed to estimate the true mean.[7] The standard deviation was employed to calculate the standard error of the mean (SEM) σ_{SEM} for this measurement via Eq. 2.

$$\sigma_{SEM} = \frac{\sigma}{\sqrt{N}} \tag{2}$$

As the objective of the repeated measurements was to achieve the most precise determination of μ possible, σ_{SEM} was employed for all DLS plots and subsequent analysis.[8] In order to facilitate comparison between the various types of vesicles, the mean diameter

increase resulting from the addition of RNA $\mu_{increase}$ was calculated for each RNA addition by subtracting the mean peak size of the measurement taken prior to RNA addition μ_{before} from the mean peak size following RNA addition μ_{after} (Equation 3).

$$\mu_{increase} = \mu_{after} - \mu_{before} \tag{3}$$

In order to calculate the standard error of the mean of the size increase $\sigma_{SEM,increase}$ from the respective measurement SEMs, the error propagation was calculated using equation 4.[8]

$$\sigma_{SEM,increase} = \sqrt{\sigma^2_{SEM,after} + \sigma^2_{SEM,before}} \tag{4}$$

These SEMs are presented in the scatter part of the DLS plots (derived from two to four individual independent replicates). The symbol n denotes independent replicates, performed on different days. For each DLS replicate, we acquired 3 technical repeats (each repeat resulting in a mean size value) and used their average as the replicate value for statistical inference. For confocal imaging of the SecAntiGami on GUVs, the measured intensities were averaged per GUV and these averages were used as the replicate value for statistical inference. The Welch's t-test was used to infer significance the cut-off p-value for significance set at 0.01.

GUV preparation

Giant unilamellar vesicles (GUVs) were prepared using the electroformation method. The lipid composition for the "Biotin-GUVs" was as follows: 5 mol% DSPE-PEG(2000)-Biotin (referred to as Biotin-PE, throughout this work), 94 mol% DOPC and 1 mol% ATTO 488-PE (for MGA SecAntiGami) or ATTO 647-PE (for BrA SecAntiGami). The "DOPC-GUVs" were composed of 99 mol% DOPC and 1 mol% ATTO 488-PE. The lipid mixtures were prepared at a concentration of 5 g/L. In detail, the conductive side of a cleaned indium tin oxide coated glass slide (ITO slide; Visiontek Systems Ltd.) was coated with 40 µL

of the lipid mixture and dried under vacuum for 30 min. A rubber O-ring with vacuum grease was applied to the lipid-coated surface. The slide was then placed into a Nanion Vesicle Prep Pro device (Nanion) and the ring was filled with 600 mM sucrose solution. The chamber was sealed with an additional ITO slide (conductive side facing to the filled ring) and the preinstalled standard protocol for GUV formation consisting of three phases was used (Initiation phase: 180 s, 3 V, 5 Hz, 37 °C, Main phase: 7380 s, 3 V, 5 Hz, 37 °C, Detaching phase: 7680 s, 0 V, 5 Hz, 37 °C; Parameters are changed linearly over time from one step to the next.). Subsequently, the GUVs were collected and stored at 4 °C for a maximum of one week.

Preparation of GFP-functionalized GUVs

GFP functionalization was done using His-tag GFP (SinoBiological, Cat. No. 13105-S07E) and 18:1 DGS-NTA(Ni) lipids (Avanti Research™, Cat. No. 790404C). NTA GUVs were prepared using the gel-assisted hydration method as described previously in.[9] In brief, a thin layer of PVA was dried on a glass slide at 60 °C. After drying of the PVA gel, 4 µL of lipid stock (mol% 95% DOPC, 5% 18:1 DGS-NTA(Ni)) at $10\,\mathrm{g\,L^{-1}}$ was spread using a Hamilton syringe and dried for one hour at 37 °C. Next, a rubber ring was placed on top of the dried film and hydrated using 300 µL of 700 mM sucrose. The chamber was then closed using another glass slide and kept at room temperature for 1 h, after which the chamber was briefly turned around and gently tapped before harvesting of the GUVs. The harvested GUVs were then kept at 4 °C for up to 1 week. To functionalize the GUVs, 4.95 µL of GUVs were diluted to 15 µL in NTA buffer (100 mM KCl, 100 mM HEPES, 112 mM sucrose) and 3 µL freshly dissolved His-GFP stock at $0.33\,\mathrm{g\,L^{-1}}$, dissolved in 1xPBS. The GUVs were left to react with the GFP for 1 hour prior to incubation with IVT mixture, as described in the section below.

Confocal microscopy

In order to directly visualize the SecAntiGami binding under a confocal microscope, the IVT reaction was conducted in the presence of the GUVs and the malachite green/DFHBI-1T dye. In detail, the reaction mixture was prepared with one part ATTO 488 or ATTO 647-PE-labelled GUVs and ten parts of a 1.1x IVT mix. The IVT mix comprised a 1.1x home-made high-yield IVT buffer (110 mM HEPES, 22 mM $Mg(OAc)_2$, 2.75 mM of each rNTP (Jena Bioscience), 1.65 mM DTT (Jena Bioscience), 1.65 mM spermidine (Sigma-Aldrich) and 73.26 mM sucrose). Additionally, the IVT mixture contained 0.5 U μL^{-1} Murine RNase Inhibitor (NEB), 2.2 %(V/V) of HiScribe T7 from the HiScribe T7 High Yield RNA Synthesis Kit, 50 µM Malachite Green (Sigma-Aldrich) or 50 µM DFHBI-1T (Lucerna Technologies), 40 nM of aptamer DNA template and 30 nM of SecAntiGami DNA template. A microscope coverslip attached to an Ibidi sticky-Slide VI 0.4 was passivated with 5 % polyvinyl alcohol (PVA, MW: 13 kDa to 23 kDa, 87-89 % hydrolyzed, Sigma-Aldrich) rinsed with nuclease-free water and dried under a nitrogen flow. Subsequently, the prepared sample was applied to the channels of the Ibidi slide. The channels were sealed with a two-component glue and incubated at 37 °C for a period of 2 h to 4 h.

Following this incubation period, the samples were visualized using a confocal laser scanning microscope (LSM 900, Carl Zeiss AG). A 20X objective (Plan-Apochromat 20×/0.8 M27, Carl Zeiss AG) was employed for image acquisition. The internal magnification was set to 2X and the pinhole was set to the optimal size for confocal imaging. The reactions were imaged at 37 °C. To achieve this, a temperature control unit, the VAHEAT from Interherence GmbH, was utilized. The reactions were imaged with two independent laser tracks: A laser at 488 nm with a laser power of 0.1-0.2 % and a laser at 640 nm with a laser power of 0.1-0.2 %.

Image analysis

For visualization purposes, the images were processed using the ImageJ software. In order to enhance visibility, the brightness and contrast were adjusted.

The unprocessed images were used for the quantitative analysis using a python code that was set up with the aid of ChatGPT and is available on the group's Github `https://github.com/Biophysical-Engineering-Group`. First, the lipid channel was normalized by division with the highest intensity value and then smoothed by Gaussian smoothing. The image of the lipid channel was segmented by multi-Otsu thresholding into the GUV regions. A morphological filter was employed to remove objects smaller than 1000 pixels, while binary filling was utilized to highlight the edges of the GUVs. Subsequently, connected objects are labeled and presented to the researcher in an interactive interface. This offers the chance to only select GUVs which were not already analyzed in a different image, are imaged in focus and are not deformed or aggregated too much to be able to ensure a good surface intensity measurement. To ensure traceability, all selections of the researcher are automatically logged. The selected GUV areas are eroded and dilated by 2 pixels and the two product areas are subtracted from each other to gain a 4 pixel thick ring-like shape at the GUV edge (found by the lipid image). The intensities of this ring on the RNA image are measured. The mean of all pixels of the image was used as the background intensity and subtracted from the mean of the ring pixels. Note that, for the Broccoli SecAntiGami no intensity correction was performed as the background intensity was = 0 (Figure S11).

Plate reader assay for fluorescence monitoring of SecAntiGami folding

Folding of the 4KL and 1KL SecAntiGami constructs was assessed in a custom transcription buffer at 37 °C (see Methods: Confocal microscopy) using a plate reader assay that detected MGA folding within each sample. Technical replicates were prepared on the same plate

(n=1) and measured in parallel. Prior to loading, microwells were passivated with PVA for 5 min, rinsed with RNase-free water, and filled with 40 µL reaction mixtures containing 30 nM DNA template, 1 µL RNase inhibitor, transcription buffer, and 50 µM Malachite Green (Sigma-Aldrich). Fluorescence was recorded using a Tecan Spark plate reader in bottom-reading mode with a transparent bottom plate, with excitation at 620 ± 10 nm, emission wavelength 660 ± 10 nm and a gain setting of 85.

Supplementary Tables

All template sequences used in the paper are also documented in Supplementary file 1 (xlsx).

Table S1: Sequences of DNA templates

Table S1: Sequences of the DNA templates. Bold characters indicate the binding sites for the PCR primers.

Sample	DNA sequence
SecAntiGami-4KL	**GGG CAT TCT AAT ACG ACT CAC TAT A**GG GAG ATA TGG GGC TGG CCA CGA ACC CGA TAC GTG GCT AGC GGG CTT TCG AGT CCG ATG CTG ACG AAC CCG ATA CGT CAG TAT CTC CTG CCA ACT TGC CAG GCG GGA CAA GAG TAA CCG TTC AAC TTT TGC CCG TAT CTC CCT AAT GTG GCT AGG GGT CAA GAA CGG AGA CTC CTG ACT CCA AAG GCA AGA TGG GGT CCA CTG GTA CGA ACC CGA TAC GTA CCG GTG CAG CGT TCG CGT TGG CCT TGA ACG AAC CCG ATA CGT TCA GGG CAG CCA TAT TAC TGC AAG AGG ATC CCG ACT GGC GAG AG**C CAG GTA ACG AAT GGA TCC TCT G**
SecAntiGami-1KL	**GGG CAT TCT AAT ACG ACT CAC TAT A**GG GAG ATA TGG GGC TGG CCA CGT TCG CGT GGC TAG CGG GCT TTC GAG TCC GAT GCT GAC GTT CGC GTC AGT ATC TCC TGC CAA CTT GCC AGG CGG GAC AAG AGT AAC CGT TCA ACT TTT GCC CGT ATC TCC CTA ATG TGG CTA GGG GTC AAG AAC GGA GAC TCC TGA CTC CAA AGG CAA GAT GGG GTC CAC TGG TAC GTT CGC GTA CCG GTG CAG CGT TCG CGT TGG CCT TGA ACG AAC CCG ATA CGT TCA GGG CAG CCA TAT TAC TGC AAG AGG ATC CCG ACT GGC GAG AG**C CAG GTA ACG AAT GGA TCC TCT G**

Broccoli SecAntiGami	**GGG CAT TCT AAT ACG ACT CAC TAT A**GG GAG ATA TGG GGC TGG CCA CGA AGT CTA GAC GTG GCT AGC GGG CTT TCG AGT CCG ATG CTG ACG AAG TCT AGA CGT CAG TAT CTC CTG CCA ACT TGC CAG GCG GGA CAA GAG TAA CCG TTC AAC TTT TGC CCG TAT CTC CCT AAT GTG GCT AGG GGT CAA GAA CGG AGA CTC CTG ACT CCA AAG GCA AGA TGG GGT CCA CTG GTA CGA AGT CTA GAC GTA CCG GTG CAG CGT TCG CGT TGG CCT TGA ACG AAG TCT AGA CGT TCA GGG CAG CCA TAT TAC TGC AAG AGG AGA CGG TCG GGT CCA GTT CGC TGT CGA G**TA GAG TGT GGG CTC CTC TG**
Biotin-KL	**GGG CAT TCT AAT ACG ACT CAC TAT A**GG ACC GTC AGA GGA CAC GGT TAA AAA GTC CTC TTT AAA CAC TAT CAA ATA AGC CTG CGT CTG GTG GGT ACG AAA TCG GGA CG**T GCC CAC TAG ACG TAG GC**
V1-KL	**GGG CAT TCT AAT ACG ACT CAC TAT A**GG GAA TGA TGA CTC ACC GTC AGA GGA CAC GGT TAA AAA GTC CTC TCG ACC GTC ATT CTC GCC TGC GTC TGG TGG GTA CGA AAT CGG GAC G**TG CCC ACT AGA CGT AGG C**
V2-KL	**GGG CAT TCT AAT ACG ACT CAC TAT A**GG ACC GTC AGA GGA CAC GGT TAA GGC CTG CGT CTG GTG GGT ACG AAA TCG GGA CGT GCC CAC TAG A**CG TAG GCC ACA AAA AGT CCT C**
V3-KL	**GGG CAT TCT AAT ACG ACT CAC TAT A**GG AAT GTC ATT CTC TTT GGA CCG TCA GAG GAC ACG GTT AAA AAG TCC TCT TTT GCC TGC GTC TGG TGG GTA CGA AAT CGG GAC GTG CCC ACT AGA CG**T AGG CGG GAA TGA TGT TCC**

N-Biotin-KL	**GGG CAT TCT AAT ACG ACT CAC TAT A**GG ACC GTC AGA CCA CAA AAA AAA AAA GTC CTC TTT AAA CAC TAT CAA ATA AGC CTG CGT CTG GTG GGT ACG AAA TCG GGA CG**T GCC CAC TAG ACG TAG GC**
Biotin-KL2 (used with broccoli SecAntiGami)	**GGG CAT TCT AAT ACG ACT CAC TAT A** GGA CCG TCA GAG GAC ACG GTT AAA AAG TCC TCT TTA AAC ACT ATC AAA TAA GCC TGC GTC TGG TGG GTA CGA ACT AGA CAC G**TG CCC ACT AGA CGT AGG C**
GFP-aptamer-KL	GGG CAT TCT AAT ACG ACT CAC TAT AGG AGC TTC TGG ACT GCG ATG GGA GCA CGA AAC GTC GTG GCG CAA TTG GGT GGG GAA AGT CCT TAA AAG AGG GCC ACC ACA GAA GCT AAA AAC ACT ATC AAA TAA GCC TGC GTC TGG TGG GTA CGA AAT CGG GAC GTG CCC ACT AGA CGT AGG C

Table S2: Primer sequences

Table S2: Sequences of the PCR primers.

Direction	DNA sequence	Used for
forward	GGG CAT TCT AAT ACG ACT CAC TAT A	all DNA templates
reverse	CAG AGG ATC CAT TCG TTA CCT GG	SecAntiGami, SecAntiGami-1KL
reverse	CAG AGG AGC CCA CAC TCT A	Broccoli SecAntiGami
reverse	GCC TAC GTC TAG TGG GCA	Biotin-KL, N-Biotin-KL, V1-KL, Biotin-KL2
reverse	GAG GAC TTT TTG TGG CCT ACG	V2-KL
reverse	GGA ACA TCA TTC CCG CCT A	V3-KL

Table S3: Estimation of the expected size increase in DLS

We roughly estimated the size of the expected maximal diameter increase of Biotin-PE SUVs by addition of Biotin-KL and SecAntiGami on DLS (see Table S3). Therefore, we

used following approximations:

- The contour length of an RNA duplex is approximately 2.8 nm.[10]
- The length of a KL complex (two kissing KLs) corresponds to approx. 8 bases.[11]
- The contour length of PEG-chain of molecular weight 2000 Da is 12.7 nm.[12]
- The full turn of an RNA double helix is roughly 11 bp.[10]

Table S3: Rough calculation of the maximal expected size increase for Biotin-KL plus SecAntiGami on Biotin-PE SUVs. Note that the DLS expects spherical like particles and thus reports the size of particles as their diameter.

**Description**	**Calculation**	**Result**
SecAntiGami width (only stem)	$34\,\text{bases} \times \frac{2.8\,\text{nm}}{11\,\text{bases}}$	8.65 nm
SecAntiGami width (KLs)	$3\,\text{KLs} \times 8\,\text{bases} \times \frac{2.8\,\text{nm}}{11\,\text{bases}}$	6.11 nm
Biotin-KL length	$52\,\text{bases} \times \frac{2.8\,\text{nm}}{11\,\text{bases}}$	13.24 nm
PEG-linker max. length		12.7 nm
SUM		40.7 nm
On both sides of the SUV	SUM × 2	81.4 nm

Supplementary Figures

Figure S1: Agarose gel of PCR product

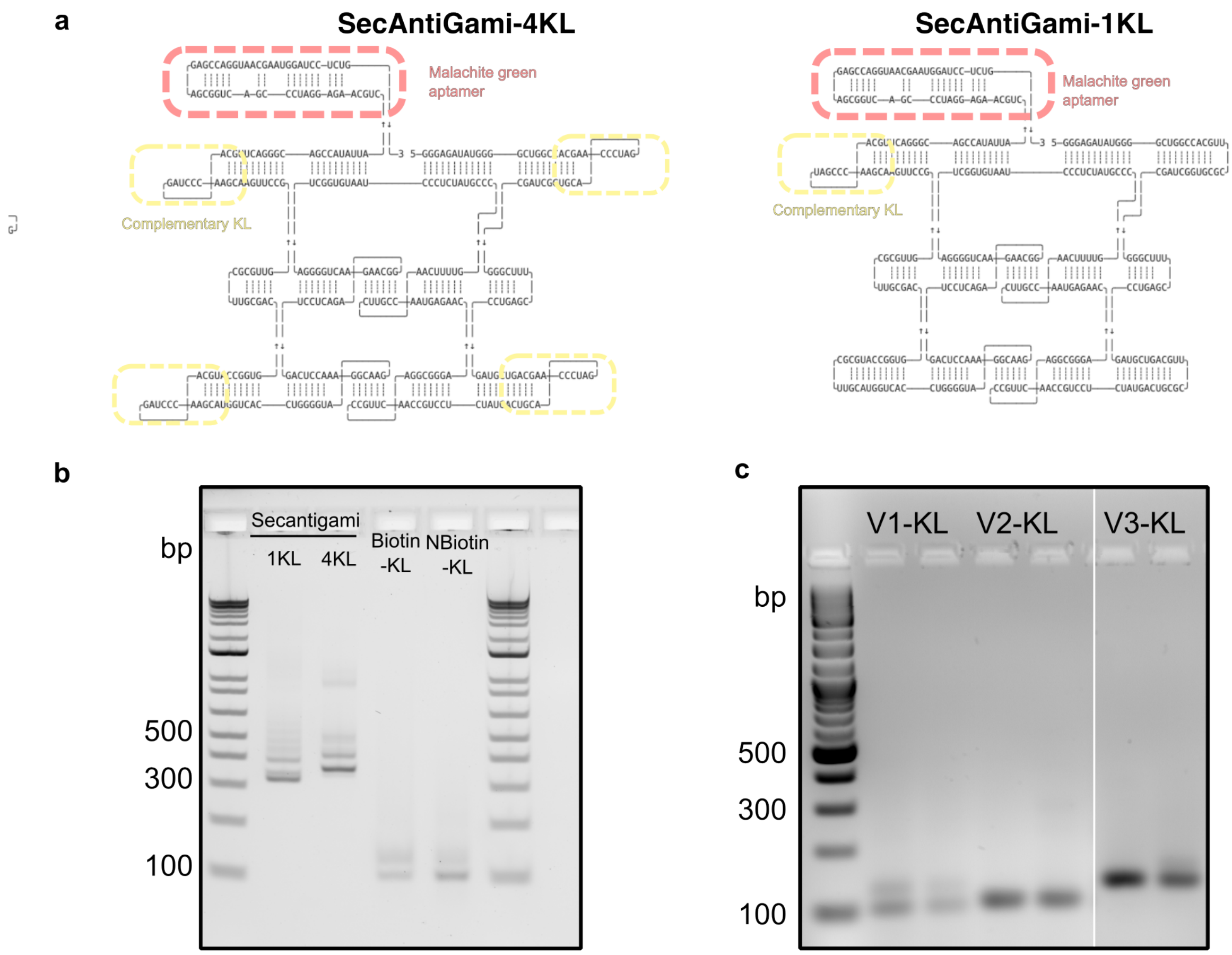

Figure S1: Agarose gel electrophoresis of PCR products used in this study. (a) Schematics showing the SecAntiGami-4KL and SecAntiGami-1KL secondary structures. **(b)** Agarose gel (2 %), of SecAntiGami-4KL, SecAntiGami-1KL, Biotin-KL and N-Biotin-KL (structures of the Biotin-KL and N-Biotin-KL are shown in Figure S8). The precast gel (E-Gel™ 2 % agarose with SYBR™ Safe) was run using the E-Gel™ Power Snap Electrophoresis Device. Using the default Sybr Safe 2 % agarose protocol for 22 min. E-Gel™ 1 kb Plus DNA-ladder is shown on the left and right side of the gel. **(c)** Agarose gel (3 %) of the PCR products of the Biotin-KL variants used in the Figures 3 and 4. The gel was run at 90 V for approximately 2 h and stained using MIDORI Green Advance at 1X concentration in the gel, additionally 4 µL of the stock solution was added to the + pole before starting the run. Both gels were imaged using the ChemiDoc™ MP Imaging System. Note that in (c) 2 lanes were cropped out of the gel for clarity.

Figure S2: PAGE gel of transcription product

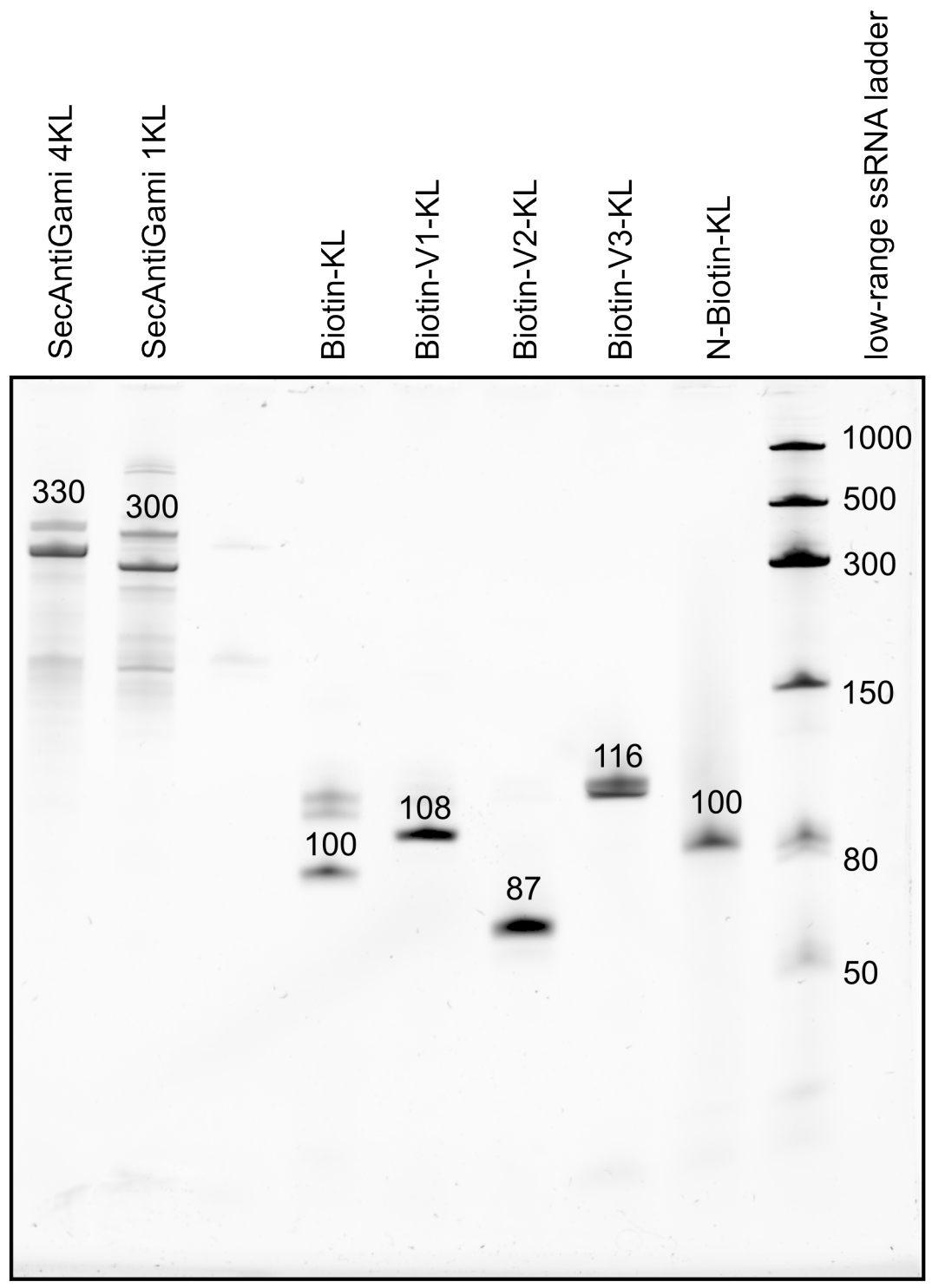

Figure S2: Polyacrylamide gel electrophoresis of transcription products used in this study. Precast Novex™ TBE-Urea Gel, 6% (Cat. No. EC6865BOX) was run at 180 V in 1 x TBE until the running front reached the last third of the gel (approx. 90 min) and stained using GelRed (30 µL in 100 mL in 1xTBE). 500 ng of purified RNA was added per condition, Low Range ssRNA Ladder (NEB, Cat. No. N0364S) is shown on the right for reference. The expected size of the transcription products is shown in the corresponding lanes. The main bands consistently appear at the expected sizes.

Figure S3: Fluorescence monitoring of SecAntiGami folding in bulk

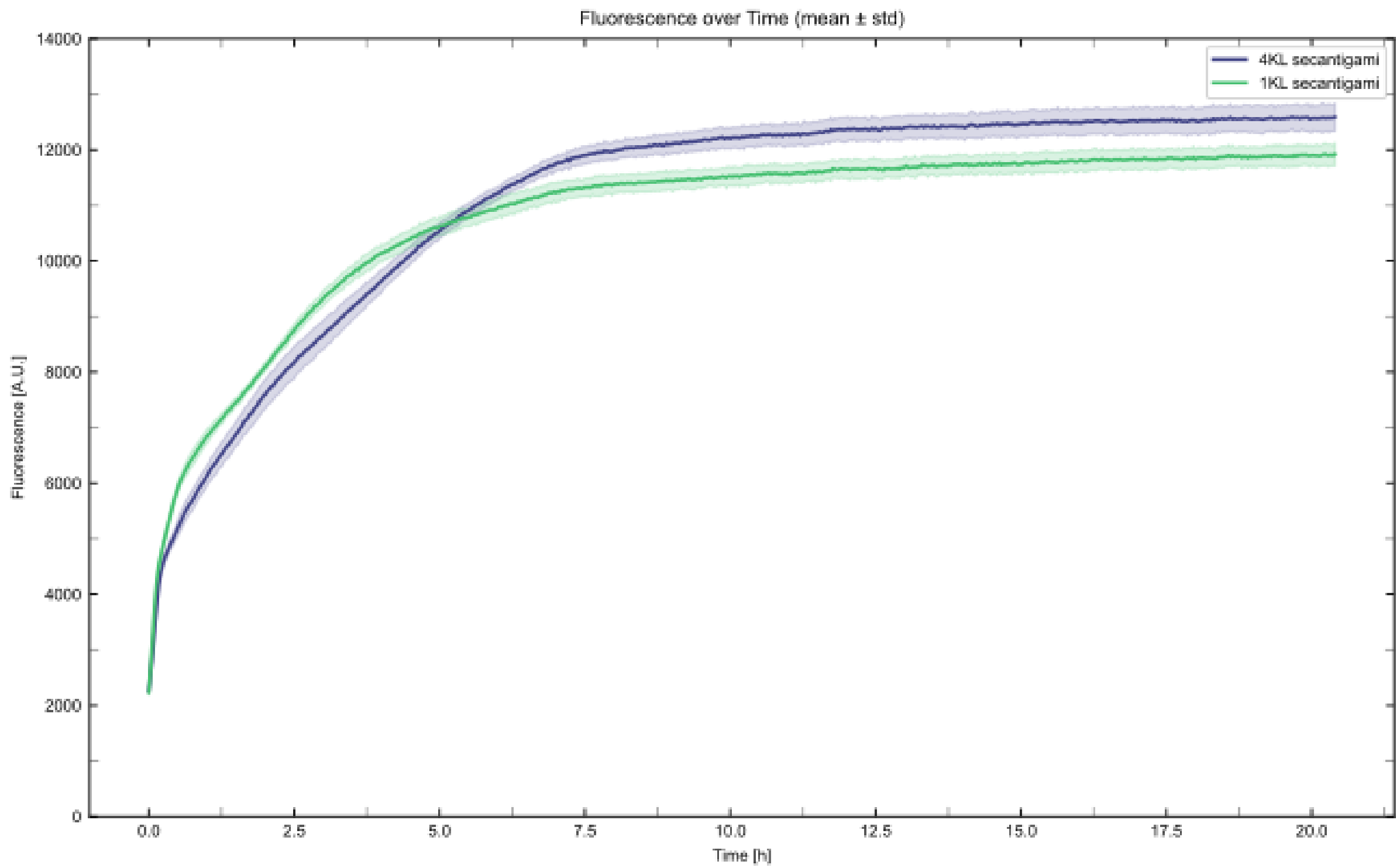

Figure S3: Fluorescence monitoring of the transcription and folding of 4KL and 1KL SecAntiGami constructs in bulk. Emission of the MGA in the SecAntiGami was monitored on the plate reader (see Materials & Methods) overnight. For both the 1KL (green) and 4KL (blue) SecAntiGami the fluorescence increases until approximately 8 h, confirming the correct folding of the origami. Data are presented with the mean (solid lines) and standard deviation (shaded regions) from technical triplicates on the same plate.

Figure S4: AFM imaging of the SecAntiGami

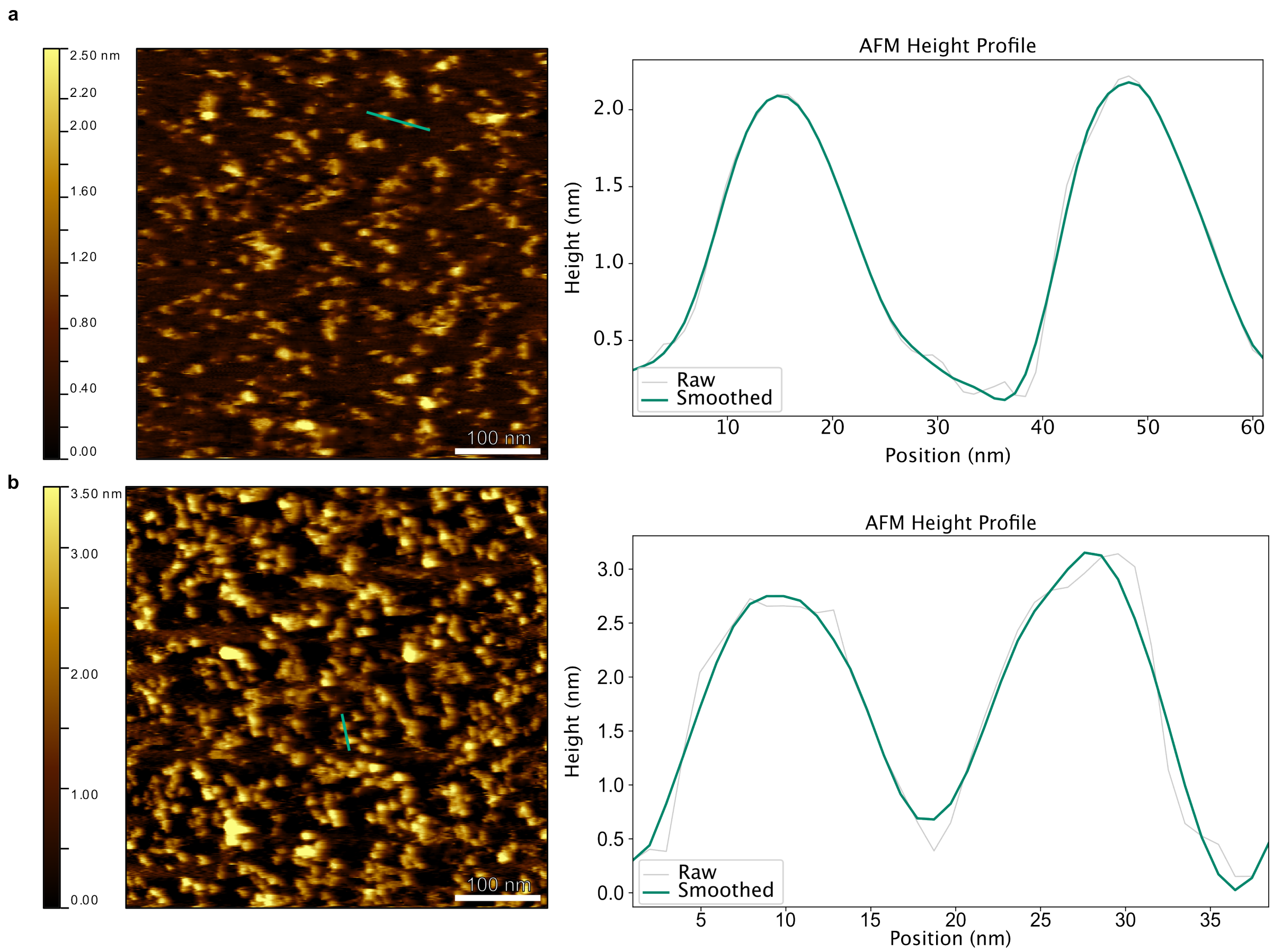

Figure S4: AFM imaging of the SecAntiGami. (a-b) Show two AFM images of the SecAntiGami after transcription. The left side shows the processed image and the right side the measured height profiles. From the width at half-maximum of the AFM height profile, we estimate the origami to be about 11 nm to 12 nm. This matches the expected dimensions shown in Figure 2, excluding the kissing loops, which are too flexible to be resolved by AFM. Scale bars, 100 nm.

Figure S5: Agarose gel of transcription product

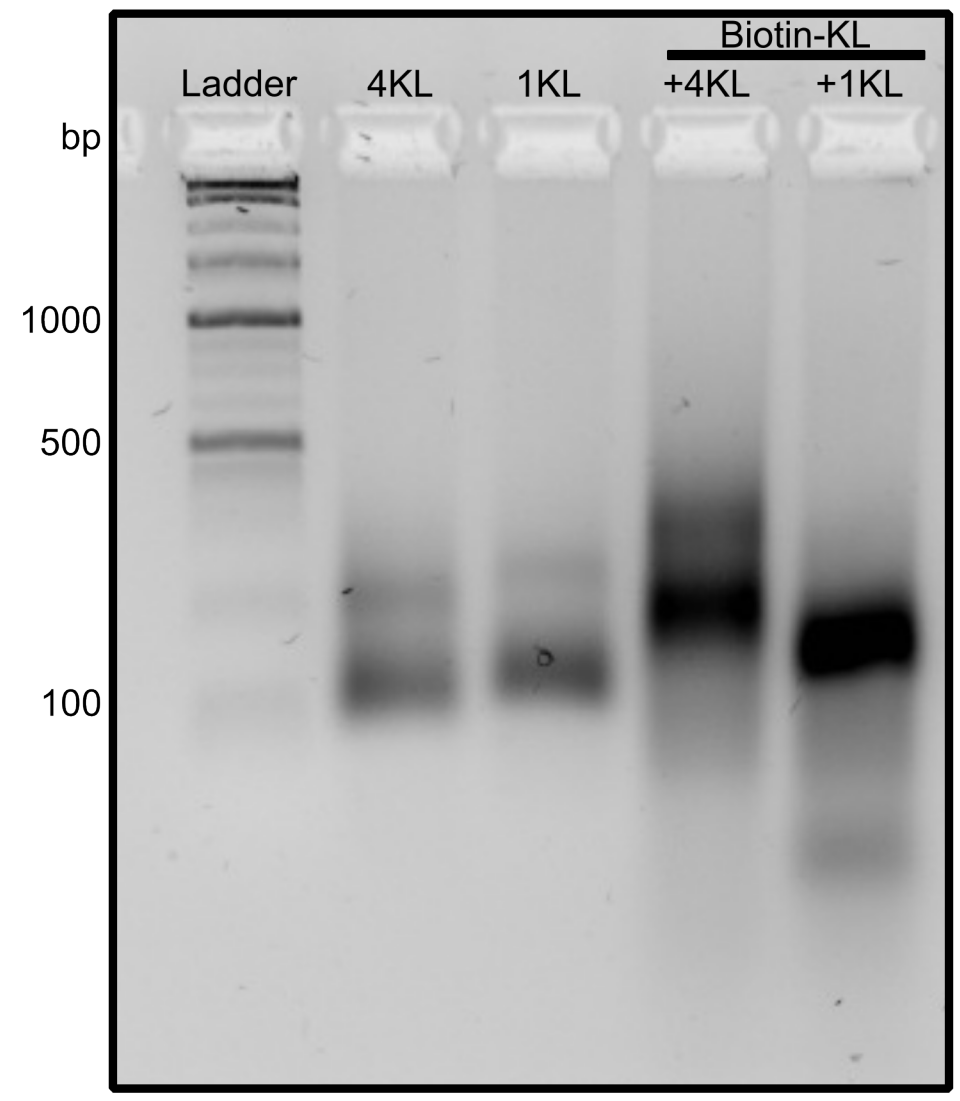

Figure S5: AGE analysis of RNA for 4KL- and 1KL-SecAntiGami with and without Biotin-KL. Agarose gel (3 %) in 1X TBE and 5 mM $Mg(OAc)_2$. The gel was run for 90 min at 85 V and stained with gel red (1X). Roughly 500 ng of RNA was loaded per lane. Samples from left to right: 1 kb Plus DNA ladder (NEB), 4 KL SecAntiGami, 1 KL SecAntiGami, 4 KL SecAntiGami + Biotin KL, 1 KL SecAntiGami + BiotinKL.

Figure S6: Sequence interferences between biotin aptamer and other fluorescent aptamers

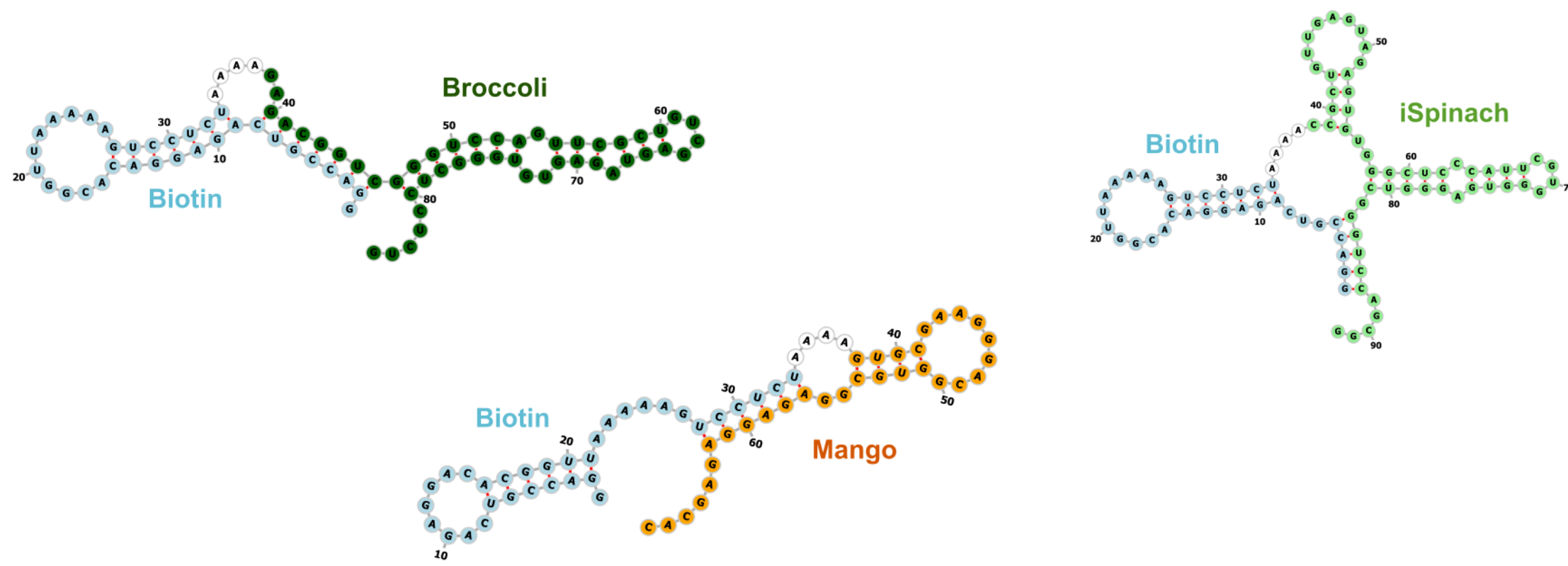

Figure S6: Complementarity of biotin aptamer sequence with fluorescent aptamer sequences. Minimum Free Energy (MFE) structures of biotin aptamer sequence concatenated with either Broccoli, iSpinach or Mango aptamers. Between the two aptamers, a linker of 4 adenosine is added. The structures are visualized with Forna. The folding predictions are done with default parameters on ViennaRNA RNAfold Webserver.

Figure S7: Sequence interferences between used aptamers and kissing loops

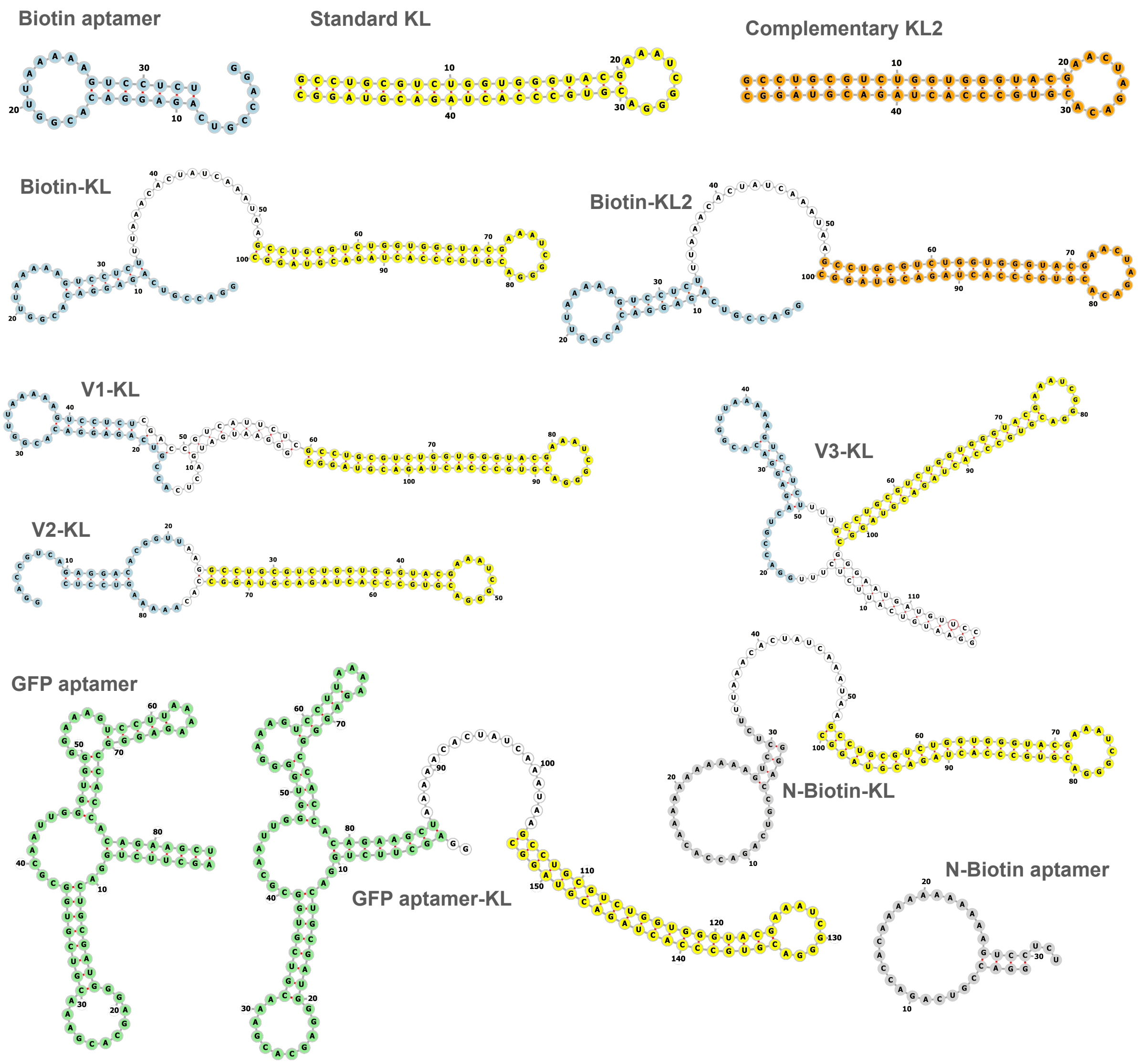

Figure S7: Predicted effect of the appended kissing loop on aptamer folding. Minimum free energy secondary structures of the biotin aptamer and variants V1-V3 (lightblue), the negative control N-Biotin aptamer (gray) and the GFP binding aptamer (green) with and without the respective KL stem-loop (yellow and orange). The aptamer secondary structure is preserved in all cases, indicating that the orthogonally designed KL does not perturb the predicted aptamer fold. Since the variants of the Biotin-KL V1-V3 were designed to be in different orientations, small deviations from the original biotin aptamer structure are expected. Structures visualized with forna. The folding predictions are done with default parameters on ViennaRNA RNAfold Webserver.

Figure S8: Diameter increase on DLS with one-armed SecAntiGami and knock-out biotin aptamer

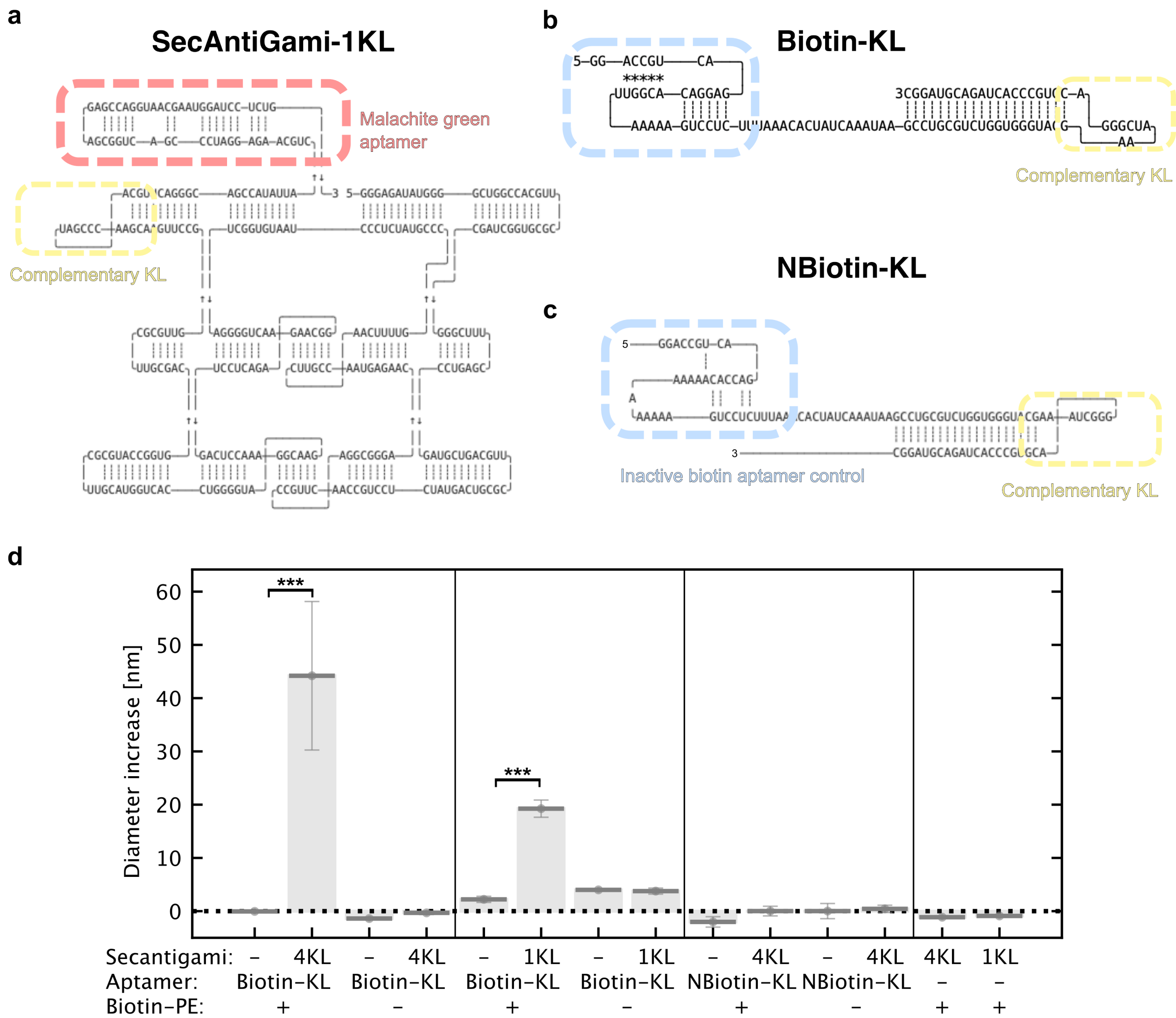

Figure S8: DLS experiments for SecAntiGami-1KL and N-Biotin-KL. (a-c) Blueprint of the SecAntiGami-1KL, Biotin-KL and N-Biotin-KL, respectively. **(d)** Diameter increase measured by DLS caused by the absence (−) of SecAntiGami or the addition of SecAntiGami with only 1KL or 4KLs on SUVs with (+) or without (−) Biotin-PEG-functionalized lipids (Biotin-PE) and pre-treated with Biotin-KL aptamers or Biotin-KL aptamers with inactivated binding sequence (N-Biotin-KL). Data of 2 independent experiments (3 independent experiments for the second bar (positive control)) are shown, the bar indicates the mean of all independent repeats while the error bar shows the SEM. Significance with p-values <0.001 are marked with ***. Note that for practical reasons, another DLS device (Zetasizer Ultra, MALVERN) was used for this experiment and Figure S9, compared with all other experiments in this study, showing the reproducibility with different devices. In addition, to estimate the RNA concentrations a different NanoDrop device (NanoDrop One Microvolume UV Spectrophotometer, Thermo Scientific) and different RNA purification columns (Monarch Spin Columns S2A, NEB) were used in preparation of the experiments.

Figure S9: Diameter increase on DLS over time

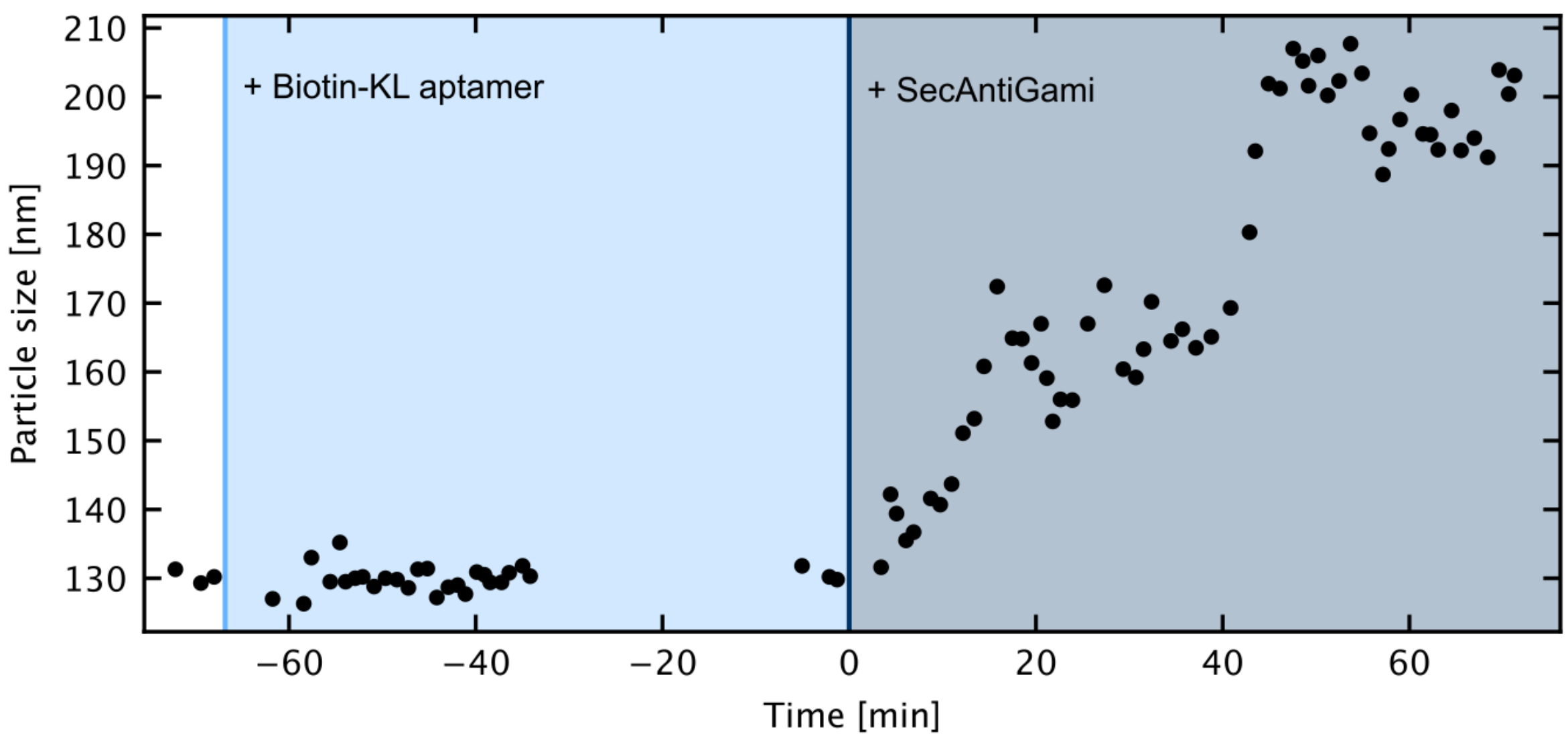

Figure S9: SUV diameter increase over time measured by DLS. SUVs are functionalized with Biotin-PEG-lipids (Biotin-PE). Biotin-KL aptamers (light blue line) and SecAntiGami with 4KLs (dark blue line) were added consecutively to the same sample. A maximal diameter increase and plateau are reached after approximately 50 min to 60 min.

Figure S10: Using the SecAntiGami to validate protein-binding aptamers

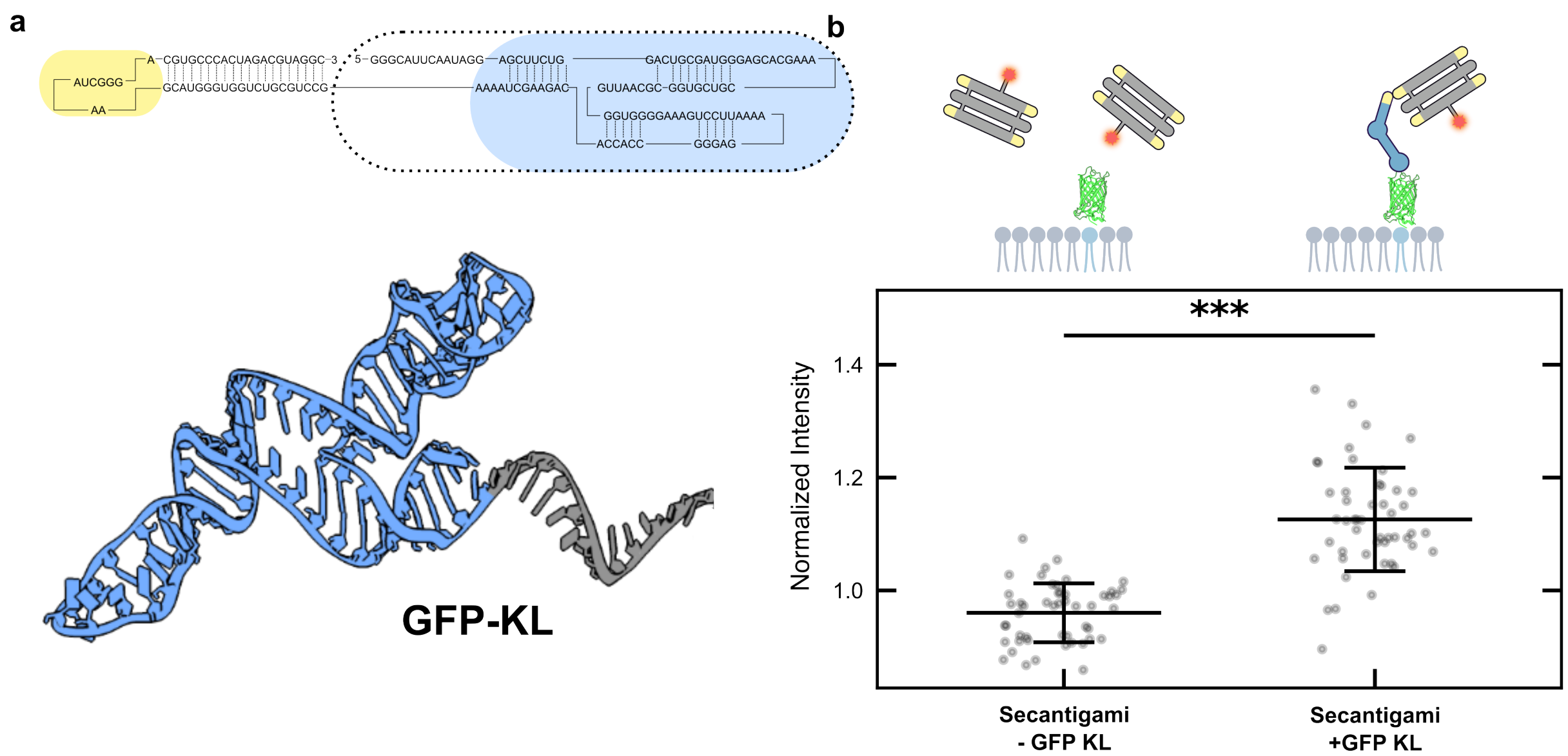

Figure S10: Using the SecAntiGami to validate protein-binding aptamers (a) Secondary structure blueprint of the GFP-KL construct. The aptamer is linked to the same KL sequence (yellow) as the biotin-KL shown in Figure 3 in the main text, and therefore compatible with the SecAntiGami. The results of a coarse-grained folding simulation in SimRNA are shown for the dotted region of each blueprint. **(b)** Quantification of fluorescence intensity on the GUV membrane for $n > 30$ GUVs, from 2 different repeats. In presence (+) and absence (−) of the GFP-KL construct (mean ± SD). ***: $p < 0.001$ (Welch's t-test). GFP was immobilized on the GUVs using NTA(Ni) lipids, as described in the Materials & Methods section.

Figure S11: Broccoli SecAntiGami validation

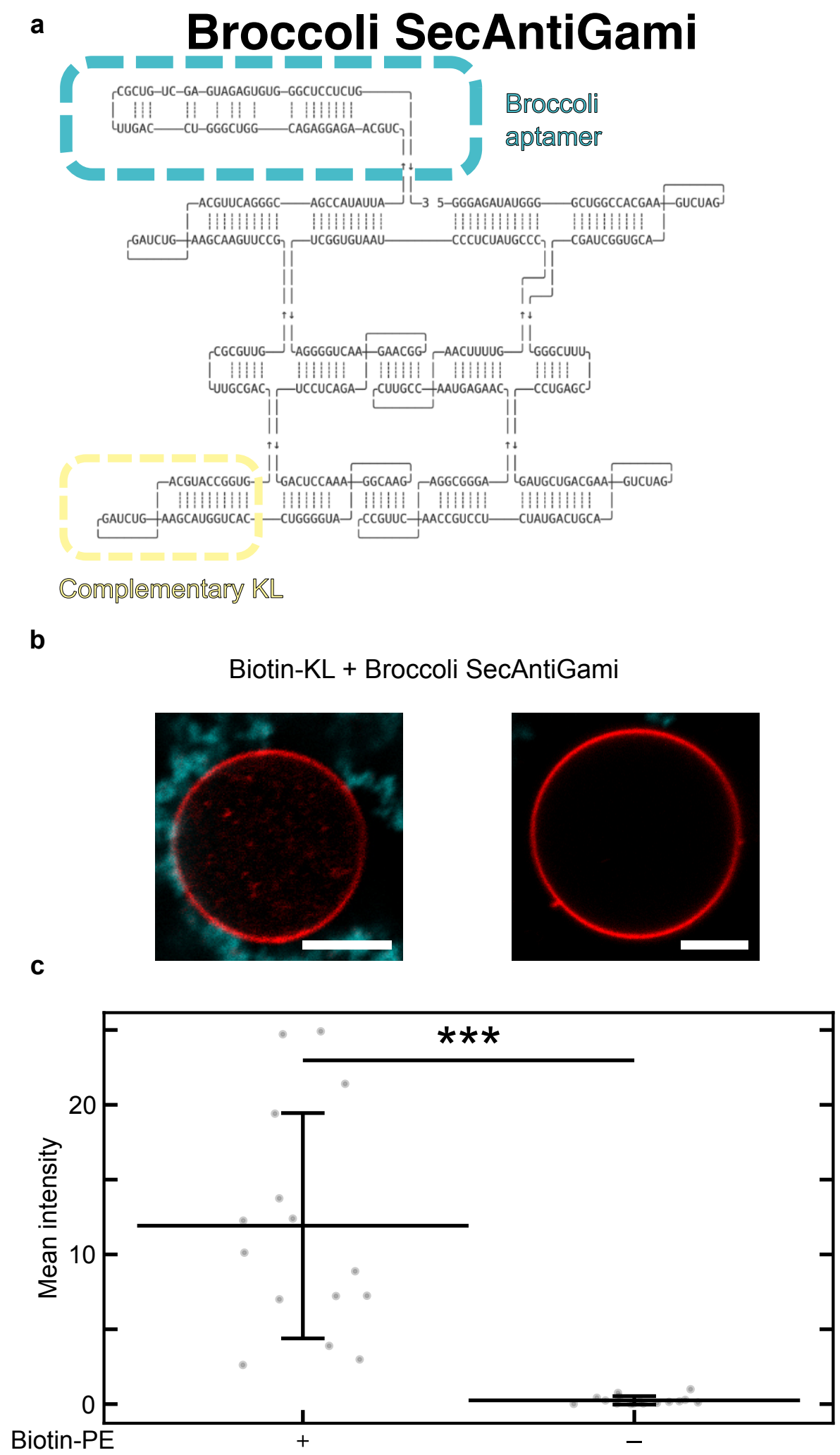

Figure S11: Design and validation of Broccoli SecAntiGami. **(a)** Blueprint of the Broccoli SecAntiGami. FLAP and KL-sequences are highlighted. **(b)** Confocal fluorescence images of representative GUVs with the Biotin-KL2 and Broccoli SecAntiGami. GUVs are shown in red and stained with 1 mol% ATTO 647 (λ_{ex}= 640 nm), SecAntiGami (shown in blue) is labeled using DFHBI-1T dye and broccoli aptamer (λ_{ex}= 488 nm), scale bars: 50 µm. Example images of GUVs (red) bound by the aggregated Broccoli SecAntiGami. **(c)** Quantification of fluorescence intensity on the GUV membrane for n=15-16 confocal images of GUVs, within a single replicate, in presence (+) and absence (−) of biotin-PE lipids for the known biotin aptamer control (mean ± SD). ***: $p < 0.001$ (Welch's t-test). Note that, no intensity correction was performed as the background intensity was zero.

Supplementary Videos

Video S1: Explanatory video animation

Video S1 is a video animation that explains the SecAntiGami concept in general terms. It was designed to make the concept broadly accessible in form of a short summary.

Supplementary References

(1) Geary, C.; Grossi, G.; McRae, E. K. S.; Rothemund, P. W. K.; Andersen, E. S. RNA origami design tools enable cotranscriptional folding of kilobase-sized nanoscaffolds. *Nature Chemistry* **2021**, *13*, 549–558.

(2) Lorenz, R.; Bernhart, S. H.; zu Siederdissen, C. H.; Tafer, H.; Flamm, C.; Stadler, P. F.; Hofacker, I. L. ViennaRNA Package 2.0. *Algorithms for Molecular Biology* **2011**, *6*, 26.

(3) Kerpedjiev, P.; Hammer, S.; Hofacker, I. L. Forna (force-directed RNA): Simple and effective online RNA secondary structure diagrams. *Bioinformatics* **2015**, *31*, 3377–3379.

(4) Gruber, A. R.; Lorenz, R.; Bernhart, S. H.; Neuböck, R.; Hofacker, I. L. The Vienna RNA Websuite. *Nucleic Acids Research* **2008**, *36*, W70–W74.

(5) Nečas, D.; Klapetek, P. Gwyddion: an open-source software for SPM data analysis. *Central European Journal of Physics* **2012**, *10*, 181–188.

(6) Wilson, C.; Nix, J.; Szostak, J. Functional Requirements for Specific Ligand Recognition by a Biotin-Binding RNA Pseudoknot. *Biochemistry* **1998**, *37*, 14410–14419, PMID: 9772167.

(7) Livingston, E. H. The mean and standard deviation: what does it all mean? *Journal of Surgical Research* **2004**, *119*, 117–123.

(8) Slama, S. *Experimentalphysik kompakt für Naturwissenschaftler*, 2nd ed.; Springer Spektrum Berlin, Heidelberg, 2020; chapter 1.

(9) Weinberger, A.; Tsai, F.-C.; Koenderink, G.; Schmidt, T.; Itri, R.; Meier, W.; Schmatko, T.; Schröder, A.; Marques, C. Gel-Assisted Formation of Giant Unilamellar Vesicles. *Biophysical Journal* **2013**, *105*, 154–164.

(10) Zhang, C.; Fu, H.; Yang, Y.; Zhou, E.; Tan, Z.; You, H.; Zhang, X. The Mechanical Properties of RNA-DNA Hybrid Duplex Stretched by Magnetic Tweezers. *Biophysical Journal* **2019**, *116*, 196–204.

(11) McRae, E. K. S.; Rasmussen, H. ; Liu, J.; Bøggild, A.; Nguyen, M. T. A.; Sampedro Vallina, N.; Boesen, T.; Pedersen, J. S.; Ren, G.; Geary, C.; Andersen, E. S. Structure, folding and flexibility of co-transcriptional RNA origami. *Nature Nanotechnology* **2023**, *18*, 808–817, Issue Date: July 2023.

(12) Ma, Z.; LeBard, D. N.; Loverde, S. M.; Sharp, K. A.; Klein, M. L.; Discher, D. E. TCR Triggering by pMHC Ligands Tethered on Surfaces via Poly(Ethylene Glycol) Depends on Polymer Length. *PLOS ONE* **2014**, *9*, e112292.
